# Application of Approximation Constructions with a Small Number of Parameters for the Estimation of a Rayleigh Fading Multipath Channel with Doppler Spectrum Spreading

**DOI:** 10.3390/s22093488

**Published:** 2022-05-03

**Authors:** Natalia E. Poborchaya, Alexander V. Pestryakov, Elizaveta O. Lobova

**Affiliations:** 1 Department of General Communication Theory, Moscow Technical University of Communications and Informatics, 111024 Moscow, Russia; 2 Department of Radio Equipment and Circuitry, Moscow Technical University of Communications and Informatics, 111024 Moscow, Russia; a.v.pestriakov@mtuci.ru; 3 Science and Research Department, Moscow Technical University of Communications and Informatics, 111024 Moscow, Russia; lizabeth2@mail.ru

**Keywords:** estimation, approximation, least squares method, a priori uncertainty, direct conversion receiver, SISO, MIMO

## Abstract

In this article, an algorithm for joint estimation of communication channel gains and signal distortions in a direct conversion receiver is proposed. The received signal model uses approximations with a small number of parameters to reduce the computational complexity of the resulting algorithm. The estimation algorithm is obtained under the assumption of a priori uncertainty about the characteristics of the communication channel and noise distribution using the linear least squares method. Estimation is performed first by the test sequence, then by the information symbols obtained after detection. In addition, an analysis of the noise immunity of quadrature amplitude modulation (QAM) signal reception is carried out using different approximating structures in the estimation algorithm for systems with a single transmitting and receiving antenna (SISO) and for systems with multiple transmitting and receiving antennas (MIMO). Furthermore, this article examines the influence of the duration of the test signal, the number of sessions of its transmission, and the channel extrapolation interval on the quality of signal reception.

## 1. Introduction

The tendency of the development of modern communication systems is aimed at increasing the quantity of transmitted information. This is achieved, for example, by using high-order modulations such as 64-, 256-, 1024-QAM, OFDM, and MIMO technologies. In this case, the quality of signal reception plays an important role. One of the methods for improving the noise immunity of communication systems is the utilization of quasi-coherent reception, which can be implemented by performing high-quality synchronization and compensation of signal distortions. The solution to the problem is based on the estimation of the communication channel and the parameters of the received signal.

The simplest and cheapest reception scheme is the direct conversion procedure, which transfers the high-frequency received signal to zero frequency and forms in-phase and quadrature components. However, this method has a major drawback, which consists in the presence of distortions: the amplitude-phase imbalance between in-phase and quadrature components (IQ imbalance), frequency shift due to a mismatch between the frequencies of the received signal and heterodyne, as well as the direct current offset (DC offset) [1,2,3,4,5,6,7,8,9,10]. Perhaps the most serious problem is DC offset in the direct conversion receivers. These DC offsets are mostly generated through self-mixing the local oscillator (LO) signal and mismatch in the mixers. In direct conversion receivers, the mixer is immediately followed by low-pass filters (LPFs) and a chain of high-gain direct-coupled amplifiers that can amplify small levels of DC offset and saturate the stages that follow. The sensitivity of the receiver can be directly limited by the DC offset component of the mixer output. Thus, direct-conversion receivers require appropriate methods to remove undesired DC offsets. The DC offset of a mixer can be separated into two components, a constant and a time-varying offset. Many DC offset cancellation techniques have been reported over the past few years [9,10]. They can be divided into baseband analog and digital techniques. Most of the reported analog techniques require large off-chip capacitors, and the digital solutions require complex digital circuitry, which is typically implemented in a separate chip. A better method is the use of baseband digital signal processing (DSP) techniques for offset estimation and cancellation.

These mentioned distortions, as a rule, are not constants, but random processes, most often slowly changing in time. They also, like the channel, greatly affect noise immunity. With the transition of communication systems to ever higher frequencies, these problems are only exacerbated. Therefore, the task of creating methods and algorithms for estimating and compensating for the described distortions and parameters of the communication channel is relevant. The more accurately they are estimated, the less the probability of an error in receiving an information symbol will be since it will be possible to provide better distortion compensation. For example, this will allow the use of error-correcting codes with less redundancy or reduce the amount of information transmitted over the reverse channel.

There are Bayesian and non-Bayesian approaches to parameter estimation. Obtaining a Bayesian estimation is associated with a large number of computational difficulties. Therefore, the maximum likelihood (ML) estimation and maximum a posteriori probability (MAP) estimation are more often used. The listed methods are based on the knowledge of a priori information about noise distribution, that is, the probability distribution density (PDF) is assumed to be known. If PDF is Gaussian, then the ML estimation and MAP estimation coincide with the Bayesian estimation. If not constants, but random processes are to be evaluated, then optimal filtering is used (linear Kalman filtering, Kolmogorov-Wiener filtering, extended Kalman filtering, indirect method of nonlinear filtering) [11,12,13,14]. These methods require knowledge of the state-transition model that describes the process being evaluated or the correlation function. However, a priori information usually is incomplete and inaccurate. Therefore, the creation of detailed mathematical models leads to the loss of the advantages of optimal algorithms over heuristic ones. Thus, if information about the noise distribution is inaccurate, two approaches are mainly used: adaptive filtering [15] and a non-parametric approach based on the stochastic approximation method [16]. The use of adaptive filters leads to a significant complication of algorithms. In addition, they are usually nonlinear and are utilized approximately. This reduces the accuracy of the estimation or leads to divergence of such algorithms. In the case of applying stochastic approximation methods, almost no a priori information is required, but the models on the basis of which the filters are synthesized are less informative. Thus, the estimations are asymptotically optimal, therefore, in the transient mode, which is most important in practice, their accuracy may not be satisfactory. In addition, as a rule, most algorithms are synthesized for Gaussian noise, which often corresponds to the real situation when the central limit theorem (CLT) of probability theory is satisfied. For example, this is true if the noise is the sum of interfering influences from a large number of sources, or normalization is used. Note, that normalization is a procedure when a random process passes through a narrowband linear system and becomes Gaussian at its output. However, interference and noise cannot always be approximated by a normal distribution. For instance, The CLT is not performed if the receiver does not have a narrow band filter. This situation can be when we expand the signal bandwidth in order to increase the throughput of the communication system. Phase noise, narrowband noise, and impulse noise are also non-Gaussian random processes. Methods of signal processing in the presence of non-Gaussian noise were studied in [17,18,19,20,21,22,23]. Also, for non-Gaussian noise with a known correlation function, a Wiener filter can be used.

To estimate the constant parameters, in the situation of a priori uncertainty, the least squares method (LS) [24] and statistical averaging over time are often used. The second approach is simple but requires a large number of samples of the received signal. For example, a DC offset, considering it unchanged during the estimation time, can be found as the average value of the observed process over time.

A review of methods [25,26,27,28,29,30,31,32] for estimating the channel and signal distortion in a direct conversion receiver showed that most often, the estimation of IQ imbalance is considered, or high-precision procedures for joint estimation of channel parameters and signal distortion, such as IQ imbalance, frequency shift, and DC offset, which have a very high computational complexity [32]. Known algorithms work either according to special test sequences [29,30,31] or “blindly” [33]. However, in [33], distortions in the direct conversion receiver were not taken into account. The estimation algorithms obtained for certain test signals are not suitable for estimating parameters from the information signals obtained after symbol detection. This means that this approach requires the transmission of a test signal again. For example, in [25] the statistical properties of the channel are known, in [26] it is necessary to know the joint PDF of the signal components. In [27], joint estimation and compensation of IQ imbalance are considered. LS is used for the synthesis of the estimation algorithm. The proposed algorithms in [28] do not estimate the frequency offset and DC offset. The work [29] offers a joint estimation of IQ imbalance and channel multipliers. The algorithm has been developed for special orthogonal test sequences. The amplitude-phase imbalance, DC offset, and phase of the signal are estimated from a binary test sequence in [30,31] using a simplified ML under the assumption of additive Gaussian white noise. Phase noise and signal amplitude are not included in the consideration. The frequency offset is considered previously estimated with high accuracy and is also excluded from the estimated vector. A joint estimation of all distortions is given in [32], but it has a very high computational complexity.

For a non-stationary Rayleigh channel with a Doppler spread spectrum, an algorithm for estimating the parameters of a communication channel was proposed in [34]. This algorithm is based on linear Kalman filtering and approximation of the channel gains by a sum of quasi-harmonics with unknown amplitudes and phases. The harmonic frequencies are assumed to be known. The disadvantages of this approach are:

(1) The need to solve the problem of spectral analysis in advance, with the help of which harmonic frequencies are determined in trigonometric approximation;
(2) The complexity of the channel estimation increases with the number of quasi-harmonics;
(3) Signal distortions of the direct conversion receiver were not taken into account.

Works [35,36,37,38,39] consider algorithms for estimating the communication channel either without distortion in the direct conversion receiver or taking into account only the amplitude and phase imbalance between in-phase and quadrature components. In addition, the authors of [40] propose a regularizing algorithm for estimating the parameters of a stationary channel in the presence of a frequency shift, amplitude and phase imbalance, as well as a DC offset for SISO systems.

The aim of this article is:

(1) To reduce the computational complexity of the algorithm of joint estimation of the communication channel parameters and signal distortions in the direct conversion receiver. This algorithm must work both on test sequences of short duration and on information symbols after detection, assuming a priori uncertainty about the channel statistics and noise distribution;
(2) The analysis of the noise immunity of QAM signal reception using different approximating structures for the communication channel gains with few estimated parameters.

Table 1 presents some symbols, vectors, and matrices used in this work.

## 2. Formulation of the Problem

### 2.1. Channel Modal

In-phase and quadrature (IQ) vector components $Y_{c}(i),Y_{s}(i)\in {\mathbb{R}}^{N}$ with elements $y_{c,l}(i),y_{s,l}(i)$, l=1,2,....,N  of the N×N MIMO system after detection are described as

(1) $$Y_{c}(i)=H_{1}(i)\Theta (i)+B_{c}(i)+{\bar{\mu }}_{c}(i),\;Y_{s}(i)=H_{2}(i)\Theta (i)+B_{s}(i)+{\bar{\mu }}_{s}(i)$$

where i=1,2,......n—discrete time, $\Theta (i)\in {\mathbb{R}}^{2N}$—a column vector contained M-QAM symbols of the signal or the test signal with elements $I_{k}(i),\;J_{k}(i)$, k=1,2,....,N , $B_{c}(i)\in {\mathbb{R}}^{N},$ $B_{s}(i)\in {\mathbb{R}}^{N}$—vectors of the DC offset slowly varying in time with elements $b_{cl},b_{sl}$, ${\bar{\mu }}_{c}(i)\in {\mathbb{R}}^{N}$, ${\bar{\mu }}_{s}(i)\in {\mathbb{R}}^{N}$—noise vector with unknown probability distribution function, $E({\bar{\mu }}_{c}(i))=E({\bar{\mu }}_{s}(i))=0_{N\times 1}$, $E({\bar{\mu }}_{c}(i){\bar{\mu }}_{c}^{T}(i))=E({\bar{\mu }}_{s}(i){\bar{\mu }}_{s}^{T}(i))=\sigma_{\mu }^{2}I_{N\times N}$ E(⋅)—expected value, ${Ι}_{N\times N}$—identity matrix.

The channel matrices are $H_{1}(i),H_{2}(i):{\mathbb{R}}^{2N}\to {\mathbb{R}}^{N}$, $H_{1}(i)={\left(\begin{array}{cc} H_{c1}(i) & -H_{s1}(i) \end{array}\right)}_{N\times 2N},$ $H_{2}(i)={\left(\begin{array}{cc} H_{s2}(i) & H_{c2}(i) \end{array}\right)}_{N\times 2N}$. Matrices $H_{c1}(i),H_{s1}(i),$ $H_{c2}(i),H_{s2}(i)$, which size is N×N, have elements $H_{1c,lk}(i),H_{1s,lk}(i)$, $H_{2c,lk}(i),H_{2s,lk}(i)$ defined as

(2) $$\begin{array}{l} H_{1c,lk}(i)=h_{c,lk}(i)\text{cos}(2\pi \Delta fT_{c}i+\varphi_{l}(i))-h_{s,lk}(i)\text{sin}(2\pi \Delta fT_{c}i+\varphi_{l}(i)),\\ H_{1s,lk}(i)=h_{c,lk}(i)\text{sin}(2\pi \Delta fT_{c}i+\varphi_{l}(i))+h_{s,lk}(i)\text{cos}(2\pi \Delta fT_{c}i+\varphi_{l}(i)), \end{array}$$

(3) $$\begin{array}{l} H_{2c,lk}(i)=\gamma_{l}(h_{c,lk}(i)\text{cos}(2\pi \Delta fT_{c}i+\varphi_{l}(i)+\Delta \varphi_{l})-h_{s,lk}(i)\text{sin}(2\pi \Delta fT_{c}i+\varphi_{l}(i)+\Delta \varphi_{l})),\\ H_{2s,lk}(i)=\gamma_{l}(h_{c,lk}(i)\text{sin}(2\pi \Delta fT_{c}i+\varphi_{l}(i)+\Delta \varphi_{l})+h_{s,lk}(i)\text{cos}(2\pi \Delta fT_{c}i+\varphi_{l}(i)+\Delta \varphi_{l})). \end{array}$$

where l,k denote the number of transmitting and receiving antennas, respectively, l,k=1,2,....,N .

The parameters that are used in models (1)–(3) are presented in Table 2.

In order to organize quasi-coherent signal reception based on (1), we propose the estimation algorithm of channel matrices ${\overset{⌢}{H}}_{1}(i),\;{\overset{⌢}{H}}_{2}(i)$ and the algorithm of distortion compensation. The article considers the distortions introduced by the communication channel and a direct conversion receiver.

### 2.2. Approximation of the Channel Matrix

Since the estimation is carried out under conditions of a priori uncertainty of the channel characteristics and noise distribution, the simplest method for estimation algorithm is the method of least squares (LS). In order to reduce the number of computational operations, it is better to use a linear least squares method, which requires a linear model comprising a linear combination of the estimated parameters. Therefore, we propose to approximate the elements of the channel matrices (2), (3) as follows [41,42]:

(4) $$\begin{array}{l} H_{1c,lk}(i)=d(i)X_{1c,lk};\;H_{1s,lk}(i)=d(i)X_{1s,lk},\\ H_{2c,lk}(i)=d(i)X_{2c,lk};\;H_{2s,lk}(i)=d(i)X_{2s,lk}. \end{array}$$

where $X_{1c,lk},\;X_{1s,lk}$, $X_{2c,lk},\;X_{2s,lk}$ are vectors of approximation coefficients, d(i)—a row vector that depends on the type of approximation. For instance, $d(i)={\left(\begin{array}{ccccc} 1 & i & i^{2} & \cdots & i^{p} \end{array}\right)}_{1\times (p+1)}$ if a polynomial of the p-th order. We propose to use an approximation with one (p=0, $X_{qc,lk}=a_{qc0,lk};\;X_{qs,lk}=a_{qs0,lk}$, q=1;2) or two (p=1, $X_{qc,lk}={\left(\begin{array}{cc} a_{qc0,lk} & a_{qc1,lk} \end{array}\right)}^{T};$ $X_{qs,lk}={\left(\begin{array}{cc} a_{qs0,lk} & a_{qs1,lk} \end{array}\right)}^{T}$, q=1;2) estimated coefficients $a_{qc0,lk},a_{qc1,lk},a_{qs0,lk},a_{qs1,lk}$ to reduce the number of computational operations:

1. Polynomial approximation with p=0: d(i)=1;
2. Polynomial approximation with p=1: $d(i)=\left(\begin{array}{cc} 1 & i \end{array}\right)$;
3. Logarithmic approximation: $d(i)=\left(\begin{array}{cc} 1 & \mathrm{lg}(i) \end{array}\right)$;
4. Hyperbolic approximation: $d(i)=\left(\begin{array}{cc} 1 & 1/i \end{array}\right)$.

### 2.3. The Algorithm for Estimating Channel Gains and Signals Distortions in a Direct Conversion Receiver Using a Test Signal

This algorithm is based on a previous algorithm that was published in [41,42]. The entire time of receiving a signal of length n is divided into $n_{1}$ intervals of length $L=n/n_{1}$, in each of which a test signal of length m is transmitted once. Within each interval, the estimation of the channel gains (2), (3) is provided, as well as their extrapolation and the extraction of information symbols over time $K_{0}$. Then re-estimation (2), (3) takes place according to the received information sequence, then detection, etc.

The estimation of approximation coefficients based on LS uses the test signal and in-phase and quadrature components of the received signal $y_{c,l}(i),y_{s,l}(i)$. The algorithm processes the signal at each receiving antenna in a sliding window of length m:

(5) $${\overset{⌢}{Z}}_{1,l}={\left(D_{c}{}^{T}D_{c}\right)}^{-1}D_{c}{}^{T}{\bar{Y}}_{c,l};\;{\overset{⌢}{Z}}_{2,l}={\left(D_{s}{}^{T}D_{s}\right)}^{-1}D_{s}{}^{T}{\bar{Y}}_{s,l},\;l=1,2,\ldots ,N$$

where ${\overset{⌢}{Z}}_{q,l}={\left(\begin{array}{ccccccc} {\overset{⌢}{X}}_{qc,l1}^{T} & \cdots & {\overset{⌢}{X}}_{qc,lN}^{T} & {\overset{⌢}{X}}_{qs,l1}^{T} & \cdots & {\overset{⌢}{X}}_{qs,lN}^{T} & {\overset{⌢}{b}}_{ql} \end{array}\right)}_{(2N(p+1)+1)\times 1}^{T},\;q=1;\;2$, ${\overset{⌢}{b}}_{1l}={\overset{⌢}{b}}_{cl},{\overset{⌢}{b}}_{2l}={\overset{⌢}{b}}_{sl}$, $D_{c}={\left(\begin{array}{ccc} D_{1} & -D_{2} & \vec{1} \end{array}\right)}_{m\times (2N(p+1)+1)},D_{s}={\left(\begin{array}{ccc} D_{2} & D_{1} & \vec{1} \end{array}\right)}_{m\times (2N(p+1)+1)}$, $D_{1}=\left(\begin{array}{ccc} d(i)I_{1}(i) & \cdots & d(i)I_{N}(i)\\ d(i-1)I_{1}(i-1) & \cdots & d(i-1)I_{N}(i)\\ \vdots & \cdots & \vdots \\ d(i-m+1)I_{1}(i-m+1) & \cdots & d(i-m+1)I_{N}(i-m+1) \end{array}\right)$, $D_{2}=\left(\begin{array}{ccc} d(i)J_{1}(i) & \cdots & d(i)J_{N}(i)\\ d(i)J_{1}(i-1) & \cdots & d(i-1)J_{N}(i-1)\\ \vdots & \cdots & \vdots \\ d(i-m+1)J_{1}(i-m+1) & \cdots & d(i-m+1)J_{N}(i-m+1) \end{array}\right)$, ${\bar{Y}}_{c,l}={\left(\begin{array}{cccc} y_{c,l}(i) & y_{c,l}(i-1) & \cdots & y_{c,l}(i-m+1) \end{array}\right)}_{m\times 1}^{T}$, ${\bar{Y}}_{s,l}={\left(\begin{array}{cccc} y_{s,l}(i) & y_{s,l}(i-1) & \cdots & y_{s,l}(i-m+1) \end{array}\right)}_{m\times 1}^{T}$, $\vec{1}$ is a unit vector of size m×1, $i=m+(j-1)L;\;j=1,2,\ldots ,n_{1}$ p=0 or 1, «T» denotes transpose.

The estimation of elements of the channel matrix and the DC offset can be written as

(6) $${\overset{⌢}{H}}_{qc,lk}(i-m+s)=d(i-m+s){\overset{⌢}{X}}_{qc,lk},\;{\overset{⌢}{H}}_{qs,lk}(i-m+s)=d(i-m+s){\overset{⌢}{X}}_{qs,lk},s=1,\ldots ,m,$$

(7) $${\overset{⌢}{b}}_{cl}={\overset{⌢}{Z}}_{1l}(2N(p+1)+1,1);\;{\overset{⌢}{b}}_{sl}={\overset{⌢}{Z}}_{2l}(2N(p+1)+1,1).$$

Further, by the method of averaging over time and transmitting antennas, we obtain expressions for estimating the amplitude and phase imbalance [38].

(8) $${\overset{⌢}{\gamma }}_{l}=\frac{1}{N}\sum_{k=1}^{N}\frac{1}{m}\sum_{s=1}^{m}\sqrt{{\overset{⌢}{V}}_{c,lk}^{2}(i-m+s)+{\overset{⌢}{V}}_{s,lk}^{2}(i-m+s)},$$

(9) $$\Delta {\overset{⌢}{\varphi }}_{l}=\frac{1}{N}\sum_{k=1}^{N}\frac{1}{m}\sum_{s=1}^{m}arctg\left(\frac{{\overset{⌢}{V}}_{s,lk}(i-m+s)}{{\overset{⌢}{V}}_{c,lk}(i-m+s)}\right).$$

In (8), (9), ${\left(\begin{array}{cc} {\overset{⌢}{V}}_{c,lk}(i-m+s) & {\overset{⌢}{V}}_{s,lk}(i-m+s) \end{array}\right)}^{T}={\overset{⌢}{V}}_{lk}(i-m+s)=B^{-1}(i-m+s){\overset{⌢}{H}}_{2,lk}(i-m+s);$ ${\overset{⌢}{H}}_{2,lk}(i-m+s)={\left(\begin{array}{cc} {\overset{⌢}{H}}_{2c,lk}(i-m+s) & {\overset{⌢}{H}}_{2s,lk}(i-m+s) \end{array}\right)}^{T};$ $B(i-m+s)=\left(\begin{array}{cc} {\overset{⌢}{H}}_{1c,lk}(i-m+s) & -{\overset{⌢}{H}}_{1s,lk}(i-m+s)\\ {\overset{⌢}{H}}_{1s,lk}(i-m+s) & {\overset{⌢}{H}}_{1c,lk}(i-m+s) \end{array}\right)$.

### 2.4. Communication Channel Extrapolation and Signal Detection

The channel gains can be extrapolated over the interval of length $K_{0}$, using estimations (6), (8), (9) in the form

(10) $${\overset{⌢}{H}}_{1c,l\;k}(i+n_{0})=d(i+n_{0}){\overset{⌢}{X}}_{1c,lk};\;{\overset{⌢}{H}}_{1s,l\;k}(i+n_{0})=d(i+n_{0}){\overset{⌢}{X}}_{1s,lk}$$

(11) $$\begin{array}{l} {\overset{⌢}{H}}_{2c,l\;k}(i+n_{0})={\overset{⌢}{\gamma }}_{l}\cos(\Delta {\overset{⌢}{\varphi }}_{l}){\overset{⌢}{H}}_{1c,l\;k}(i+n_{0})-{\overset{⌢}{\gamma }}_{l}\sin(\Delta {\overset{⌢}{\varphi }}_{l}){\overset{⌢}{H}}_{1s,l\;k}(i+n_{0}),\\ {\overset{⌢}{H}}_{2s,l\;k}(i+n_{0})={\overset{⌢}{\gamma }}_{l}\cos(\Delta {\overset{⌢}{\varphi }}_{l}){\overset{⌢}{H}}_{1s,l\;k}(i+n_{0})+{\overset{⌢}{\gamma }}_{l}\sin(\Delta {\overset{⌢}{\varphi }}_{l}){\overset{⌢}{H}}_{1c,l\;k}(i+n_{0}), \end{array}$$

$n_{0}=1+(j_{1}-1)K_{0},\ldots ,j_{1}K_{0}$, $j_{1}=1,\ldots ,Q$.

The length of the extrapolation interval and the number of such intervals are related by a relation $Q=\frac{L-m}{K_{0}}$.

Then DC offset is compensated and a soft solution is found using the Zero Forcing method. Hard decisions are calculated using the minimum distance criterion between the soft decision vector for each receiving antenna and the vector of possible information symbols. If the noise has a Gaussian distribution, then this approach coincides with the maximum likelihood method.

### 2.5. The Algorithm for Estimating Channel Gains Using Information Signal

This is widely known that the communication channel changes over time. Therefore, it is necessary to refine the channel gains estimation using detected symbols. The linear LS algorithm of estimation was obtained in [41,42]:

(12) $${\overset{⌢}{X}}_{l}={\left(D^{T}D\right)}^{-1}D^{T}{\bar{Y}}_{l},\;l=1,2,\ldots ,N$$

${\bar{Y}}_{l}={\left(\begin{array}{cc} {\bar{Y}}_{c,l} & {\bar{Y}}_{s,l} \end{array}\right)}_{2m_{1}\times 1}^{T}$, ${\bar{Y}}_{c,l}={\left(\begin{array}{cccc} y_{c,l}(i_{0}) & y_{c,l}(i_{0}-1) & \cdots & y_{c,l}(i_{0}-m_{1}+1) \end{array}\right)}_{m_{1}\times 1}^{T}$, ${\bar{Y}}_{s,l}={\left(\begin{array}{cccc} y_{s,l}(i_{0}) & y_{s,l}(i_{0}-1) & \cdots & y_{s,l}(i_{0}-m_{1}+1) \end{array}\right)}_{m_{1}\times 1}^{T}$, ${\overset{⌢}{X}}_{l}={\left(\begin{array}{cccccc} {\overset{⌢}{X}}_{1c,l1}^{T} & \cdots & {\overset{⌢}{X}}_{1c,lN}^{T} & {\overset{⌢}{X}}_{1s,l1}^{T} & \cdots & {\overset{⌢}{X}}_{1s,lN}^{T} \end{array}\right)}_{(2N(p+1))\times 1}^{T},$ $D={\left(\begin{array}{cc} D_{11} & D_{12}\\ D_{21} & D_{22} \end{array}\right)}_{2m_{1}\times (2N(p+1))}$, $m_{1}\geq m$, $D_{11}=\left(\begin{array}{ccc} d(i_{0}){\overset{⌢}{I}}_{1}(i_{0}) & \cdots & d(i_{0}){\overset{⌢}{I}}_{N}(i_{0})\\ d(i_{0}-1){\overset{⌢}{I}}_{1}(i_{0}-1) & \cdots & d(i_{0}-1){\overset{⌢}{I}}_{N}(i_{0}-1)\\ \vdots & \cdots & \vdots \\ d(i_{0}-m_{1}+1){\overset{⌢}{I}}_{1}(i_{0}-m_{1}+1) & \cdots & d(i_{0}-m_{1}+1){\overset{⌢}{I}}_{N}(i_{0}-m_{1}+1) \end{array}\right)$, $D_{12}=\left(\begin{array}{ccc} -d(i_{0}){\overset{⌢}{J}}_{1}(i_{0}) & \cdots & -d(i_{0}){\overset{⌢}{J}}_{N}(i_{0})\\ -d(i_{0}-1){\overset{⌢}{J}}_{1}(i_{0}-1) & \cdots & -d(i_{0}-1){\overset{⌢}{J}}_{N}(i_{0}-1)\\ \vdots & \cdots & \vdots \\ -d(i_{0}-m_{1}+1){\overset{⌢}{J}}_{1}(i_{0}-m_{1}+1) & \cdots & -d(i_{0}-m_{1}+1){\overset{⌢}{J}}_{N}(i_{0}-m_{1}+1) \end{array}\right)$, $D_{21}=\left(\begin{array}{ccc} d(i_{0}){\overset{⌢}{V}}_{c1}(i_{0}) & \cdots & d(i_{0}){\overset{⌢}{V}}_{cN}(i_{0})\\ d(i_{0}-1){\overset{⌢}{V}}_{c1}(i_{0}-1) & \cdots & d(i_{0}-1){\overset{⌢}{V}}_{cN}(i_{0}-1)\\ \vdots & \cdots & \vdots \\ d(i_{0}-m_{1}+1){\overset{⌢}{V}}_{c1}(i_{0}-m_{1}+1) & \cdots & d(i_{0}-m_{1}+1){\overset{⌢}{V}}_{cN}(i_{0}-m_{1}+1) \end{array}\right)$, $D_{22}=\left(\begin{array}{ccc} d(i_{0}){\overset{⌢}{V}}_{s1}(i_{0}) & \cdots & d(i_{0}){\overset{⌢}{V}}_{sN}(i_{0})\\ d(i_{0}-1){\overset{⌢}{V}}_{s1}(i_{0}-1) & \cdots & d(i_{0}-1){\overset{⌢}{V}}_{sN}(i_{0}-1)\\ \vdots & \cdots & \vdots \\ d(i_{0}-m_{1}+1){\overset{⌢}{V}}_{s1}(i_{0}-m_{1}+1) & \cdots & d(i_{0}-m_{1}+1){\overset{⌢}{V}}_{sN}(i_{0}-m_{1}+1) \end{array}\right)$, ${\overset{⌢}{V}}_{ck}(i)={\overset{⌢}{\gamma }}_{l}({\overset{⌢}{J}}_{k}(i)\cos(\Delta {\overset{⌢}{\varphi }}_{l})+{\overset{⌢}{I}}_{k}(i)\sin(\Delta {\overset{⌢}{\varphi }}_{l}))$, ${\overset{⌢}{V}}_{sk}(i)={\overset{⌢}{\gamma }}_{l}(-{\overset{⌢}{J}}_{k}(i)\text{sin}(\Delta {\overset{⌢}{\varphi }}_{l})+{\overset{⌢}{I}}_{k}(i)\text{cos}(\Delta {\overset{⌢}{\varphi }}_{l}))$, k=1,2,…,N, $i_{0}=i+j_{1}K_{0}$, $j_{1}=1,\ldots ,Q$, ${\overset{⌢}{\gamma }}_{l},\Delta {\overset{⌢}{\varphi }}_{l}$—the estimation of amplitude and phase imbalance (8), (9). After estimating the extrapolation and detection are carried out again.

***Comment.*** The algorithm, described by Equation (12) can be used for refining ${\overset{⌢}{H}}_{1c,lk}(i),{\overset{⌢}{H}}_{1s,lk}(i)$ by the test signal after estimating (5)–(9).

**Figure 1:**
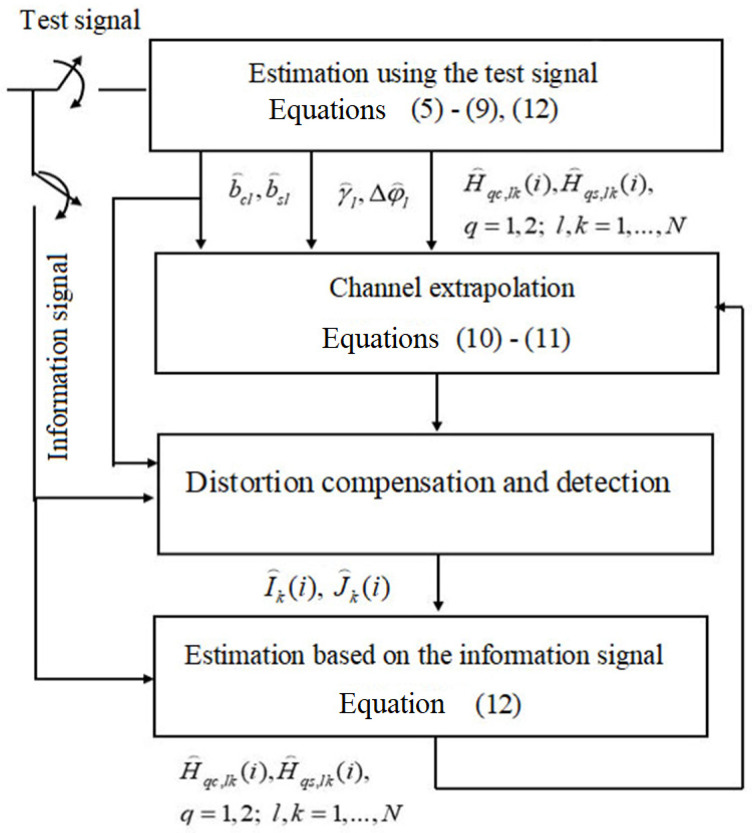
The structure of the signal processing algorithm in a MIMO communication system.

Figure 1 shows the structure of the signal processing algorithm, described by Equations (5)–(12).

## 3. Simulation Results

The analysis of the considered algorithms for estimating channel gains and signal distortions in the direct conversion receiver is carried out with the number of transmitting and receiving antennas: N=1,2,4. The multipliers $h_{c,l\;k}(i),\;h_{s,l\;k}(i)$ in the channel model (2), (3) were generated using the Jakes model as a sum of harmonics, taking into account the Doppler expansion of the signal spectrum subject to Rayleigh fading [31]. The test and information signal are modulated by M-QAM with the number of positions M=4;16;64. The phase noise $\zeta_{l}(i)$ was formed using a second-order sliding average model. The signal was received in the system without coding. The noise is Gaussian.

### 3.1. Comparison of the Proposed Algorithms with the Known Algorithm

At the first stage of simulation, the algorithms described by Equations (5) and (6) with a polynomial approximation of the order p=1 was compared with the estimation proposed in [31]. The algorithm [31] utilizes trigonometric approximation and a recurrent Kalman filter for SISO systems (N=1) in the absence of an amplitude and phase imbalance, as well as the DC offset.

The gist of the method from [31] is described below. The channel is approximated by a trigonometrical series:

$$\begin{array}{l} h_{c}(i)=\sum_{\upsilon =0}^{p}\left[A_{\upsilon }\cos(2\pi f_{\upsilon }Ti)-B_{\upsilon }\sin(2\pi f_{\upsilon }Ti)\right],\\ \;h_{s}(i)=\sum_{\upsilon =0}^{p}\left[C_{\upsilon }\cos(2\pi f_{\upsilon }Ti)-D_{\upsilon }\sin(2\pi f_{\upsilon }Ti)\right], \end{array}$$

where $A_{\upsilon },B_{\upsilon },C_{\upsilon },D_{\upsilon }$ are unknown amplitudes of harmonics, $f_{\upsilon }=F_{D}\cos\left(\frac{\pi \upsilon }{2L_{0}+1}\right),$ l=0,1,…,p, $F_{D}$ is the Doppler shift known in advance, T is a duration of the test or information signal, $L_{0}$ is a number of harmonics in the Jakes model.

Then the model of the dynamical system and the observational equations can be written as:

X(i)=X(i−1)+ζ(i), Y(i)=V(i)F(i)X(i)+μ(i),

where $X(i)={\left(\begin{array}{cccccccccccc} A_{0} & \cdots & A_{p} & C_{0} & \cdots & C_{p} & B_{0} & \cdots & B_{p} & D_{0} & \cdots & D_{p} \end{array}\right)}_{4(p+1)\times 1}^{T}$ is a vector of estimated parameters, $Y(i)={\left(\begin{array}{cc} y_{c}(i) & y_{s}(i) \end{array}\right)}^{T},$ $V(i)=\left(\begin{array}{cc} I(i) & -J(i)\\ J(i) & I(i) \end{array}\right),\;$ $\mu (i)={\left(\begin{array}{cc} \mu_{c}(i) & \mu_{s}(i) \end{array}\right)}^{T}$, ζ(i)—noise of the dynamic system, $E(\zeta (i))=0,E(\zeta (i)\zeta^{T}(i))=\sigma_{\zeta }^{2}{Ι}_{4(p+1`)\times 4(p+1)}$,

$$F(i)=\left(\begin{array}{c} \cos(2\pi f_{0}Ti)\cdots \cos(2\pi f_{p}Ti)0_{1\times (p+1)}-\sin(2\pi f_{0}Ti)\cdots -\sin(2\pi f_{p}Ti)0_{1\times (p+1)}\\ 0_{1\times (p+1)}\cos(2\pi f_{0}Ti)\cdots \cos(2\pi f_{p}Ti)0_{1\times (p+1)}-\sin(2\pi f_{0}Ti)\cdots -\sin(2\pi f_{p}Ti) \end{array}\right).$$

The estimation that is optimal according to the minimum standard deviation criterion is written as

$$\begin{array}{l} \overset{⌢}{X}(i)=\overset{⌢}{X}(i-1)+K(i)(Y(i)-V(i)F(i)\overset{⌢}{X}(i-1)),\;\\ \overset{⌢}{h}(i)={\left(\begin{array}{cc} {\overset{⌢}{h}}_{c}(i) & {\overset{⌢}{h}}_{s}(i) \end{array}\right)}^{T}=F(i)\overset{⌢}{X}(i),i=1,2,\ldots , \end{array}$$

$$\begin{array}{l} K(i)=P(i)F^{T}(i)V^{T}(i){\left[V(i)F(i)P(i)F^{T}(i)V^{T}(i)+\sigma_{\mu }^{2}{Ι}_{2\times 2}\right]}^{-1},\;\\ P(i)=\Gamma (i-1)+\sigma_{\zeta }^{2}{Ι}_{4(p+1)\times 4(p+1)},\;\Gamma (i)=P(i)-K(i)V(i)F(i)P(i). \end{array}$$

$P(i)=E(X(i)-\overset{⌢}{X}(i-1)){\left(X(i)-\overset{⌢}{X}(i-1)\right)}^{T}$—extrapolation error correlation matrix, $\Gamma (i)=E(X(i)-\overset{⌢}{X}(i)){\left(X(i)-\overset{⌢}{X}(i)\right)}^{T}$—filtering error correlation matrix.

**Figure 2:**
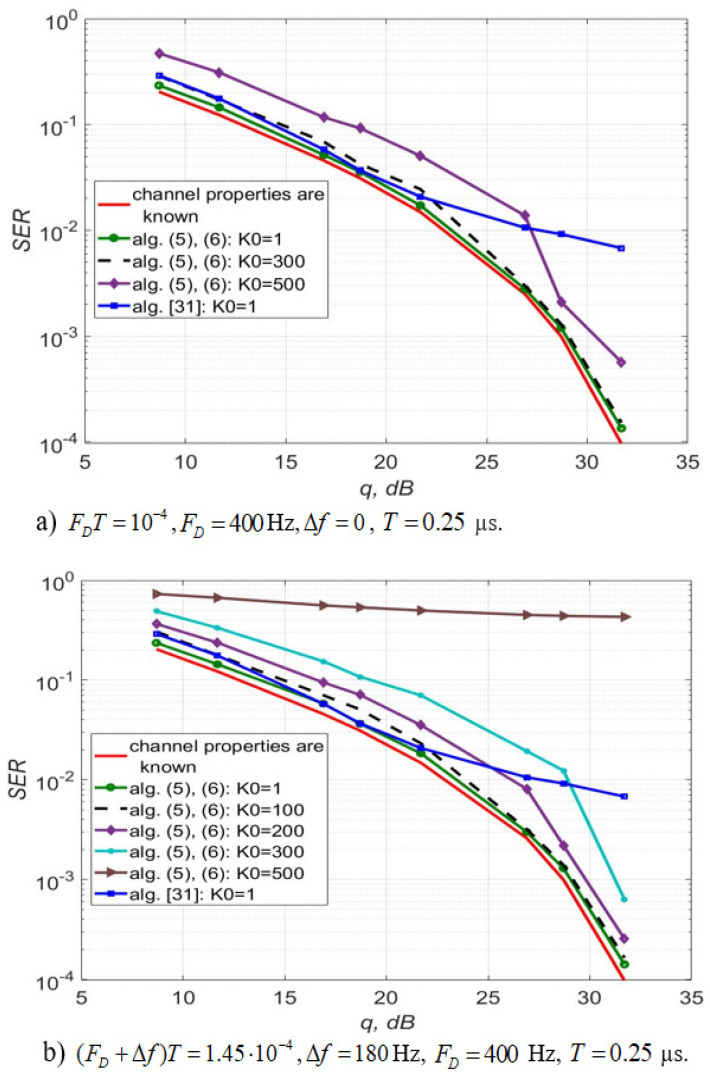
SER versus signal-noise ratio per bit for a 64-QAM signal in a channel with slow Rayleigh fading in the absence of IQ imbalance and DC offset using Equations (5) and (6) and from [31].

Figure 2 shows a symbol error rate (SER) obtained using the algorithms, described by Equations (5) and (6), with p=1 and the method [31] with p=5, $\sigma_{\zeta }^{2}=10^{-8}$, Δf=0 Hz for 64-QAM modulation of the signal and different lengths of the extrapolation interval $K_{0}$. The simulation was carried out for a slow Rayleigh fading channel. The test signal of the length m=500 was used $n_{1}=1$ times, the detection sample size is n−m=6000. The number of quasi-harmonics in the Jakes model is $L_{0}=5$, a standard deviation (SD) of the phase noise is about 1 degree, the initial phase is $\varphi_{0}=\frac{\pi }{12}$, a frequency shift Δf varies from 0 to 180 Hz. During the simulation, one sample was taken per symbol (test or information).

It can be seen that both algorithms have similar performance if the signal-noise ratio (SNR) is below 20 dB. However, when the SNR value is above 20 dB, the use of the algorithms, described by Equations (5) and (6) allows obtaining an energy gain of up to 7 dB compared to the algorithm with trigonometric approximation. In addition, the algorithms, described by Equations (5) and (6) with the channel extrapolation length $K_{0}=1$ and $K_{0}=300$ for “a)” ($K_{0}=100$ for “b)”) makes it possible to obtain almost the same noise immunity. This allows estimating the parameters of the channel using information symbols after the detection less frequently relative to the algorithm [31], for which $K_{0}=1$. Thus, the computational complexity of the signal processing algorithm is reduced. For example, for $(F_{D}+\Delta f)T=1.45\times 10^{-4}$, the number of sessions of the procedure (5), (6) is 100 times less than in [31].

The computational complexity of the considered algorithms is analyzed. It was defined as the number of addition and multiplication operations $N_{ОП}$. A detailed description of finding the approximate number of arithmetic operations is given in Table 3 for the algorithm [31] and in Table 4 for the algorithm proposed in this article with polynomial approximation and the estimation of IQ imbalance.

The calculation of the number of operations for the formation of the matrix $D_{s}{}^{T}D_{s}$ is not performed, because it contains the same elements as the matrix $D_{c}{}^{T}D_{c}$. The use of polynomial approximation leads to a decrease in computational complexity when calculating $D_{c}{}^{T}D_{c}$ since it contains repetitive elements.

Table 5 shows the computational complexity of the estimation obtained on the basis of Table 2 and Table 3 for different approximation orders.

Table 5 shows that the algorithm with a trigonometric approximation is more complicated than the methods (5), (8), (9). For example, for the first order of polynomial approximation and the signal sample size m=500, the number of arithmetic operations (5), (8), (9) is 22 times less than for the algorithm [31] with two quasi-harmonics in trigonometric approximation and 80 times less if the number of harmonics is five.

### 3.2. Analysis of Proposed Algorithms with Approximating Structures with a Small Number of Parameters

The simulation was carried out with the following values of parameters: DC offsets $b_{cl},\;b_{sl}$, amplitude imbalance $\gamma_{l}$, phase imbalance $\Delta \varphi_{l}$, and the initial random phase $\varphi_{l0}$ were formed as uniformly distributed random variables on the intervals [0, 2], [0.5, 1], $\left[-\frac{\pi }{18},\;\frac{\pi }{18}\right]$ and [−π, π], respectively, l,k=1,2,....,N. The SD of phase noise is about one degree, the total volume of the signal sample (test signal plus information signal) for one implementation is 6600 symbols, and the total number of test symbols for the entire reception time remains constant $mn_{1}=\mathrm{const}=1000$.

#### 3.2.1. SISO Systems

We consider a channel with fast Rayleigh fading $F_{D}T>10^{-3}$ in this section for SISO systems.

Model parameters are defined as $(F_{D}+\Delta f)T=1.045\times 10^{-3}$, $F_{D}=4$ kHz, Δf=180 Hz, T=0.25 µs.

**Figure 3:**
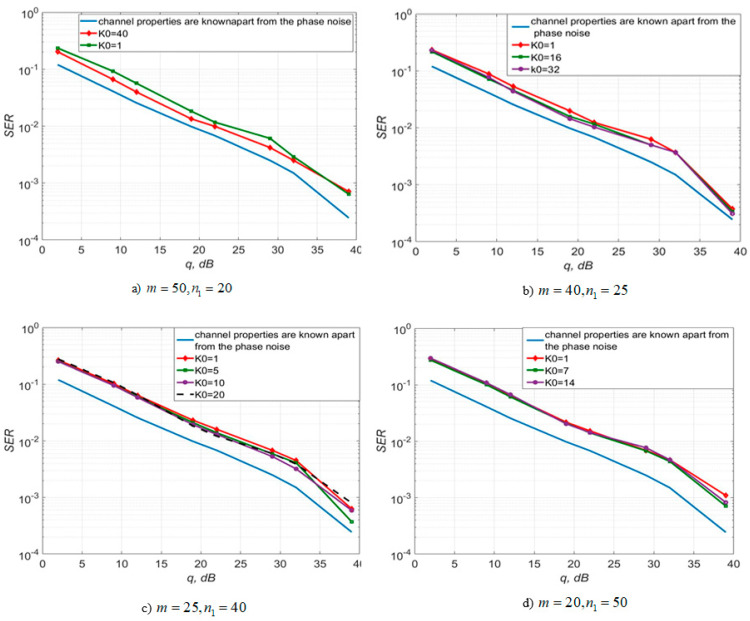
SER of 4-QAM versus SNR per bit for the algorithms, described by Equations (5)–(9) and (12) with polynomial approximation of the first order for different values of m and $K_{0}$.

**Figure 4:**
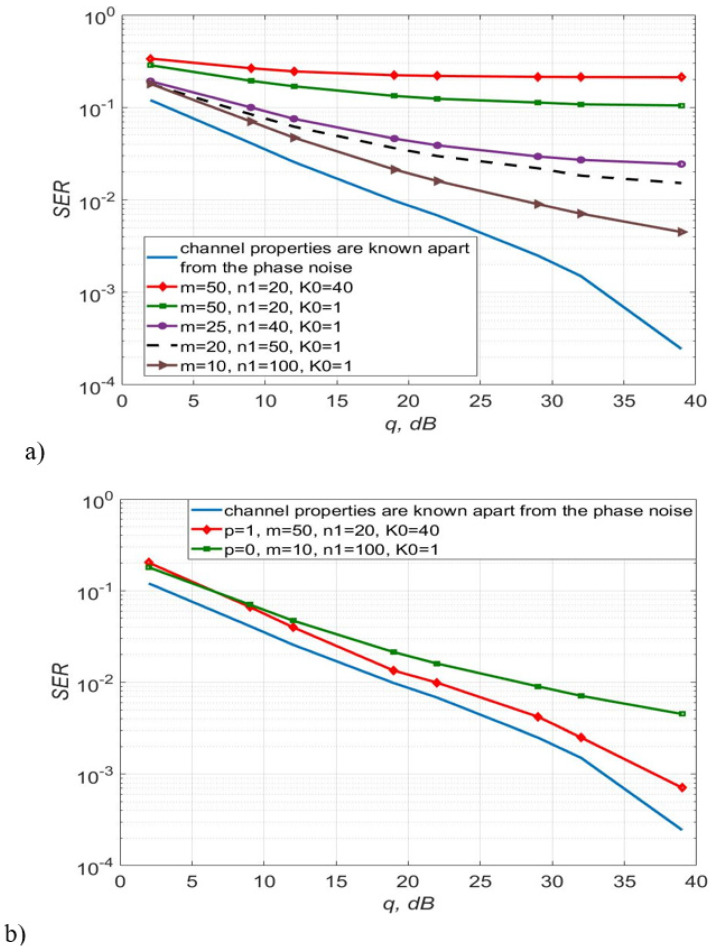
SER of 4-QAM versus SNR per bit for the algorithms, described by Equations (5)–(9) and (12) with polynomial approximation of the zero order—(**a**), zero and first orders—(**b**).

**Figure 5:**
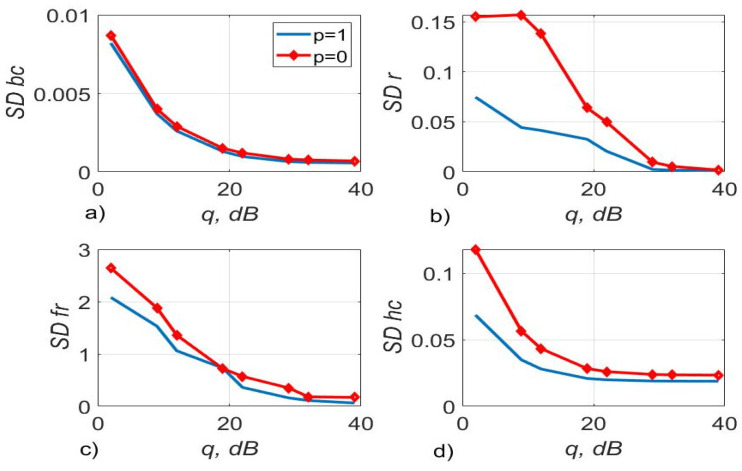
The SD of estimating DC offset (SD bc)—(**a**), amplitude imbalance (SD r)—(**b**), phase imbalance (SD fr) (deg)—(**c**) and channel gains (SD hc)—(**d**) versus SNR per bit, the test signal was modulated by 4-QAM and the algorithms, described by Equations (5)–(9) and (12) with p=1, p=0 was used.

**Figure 6:**
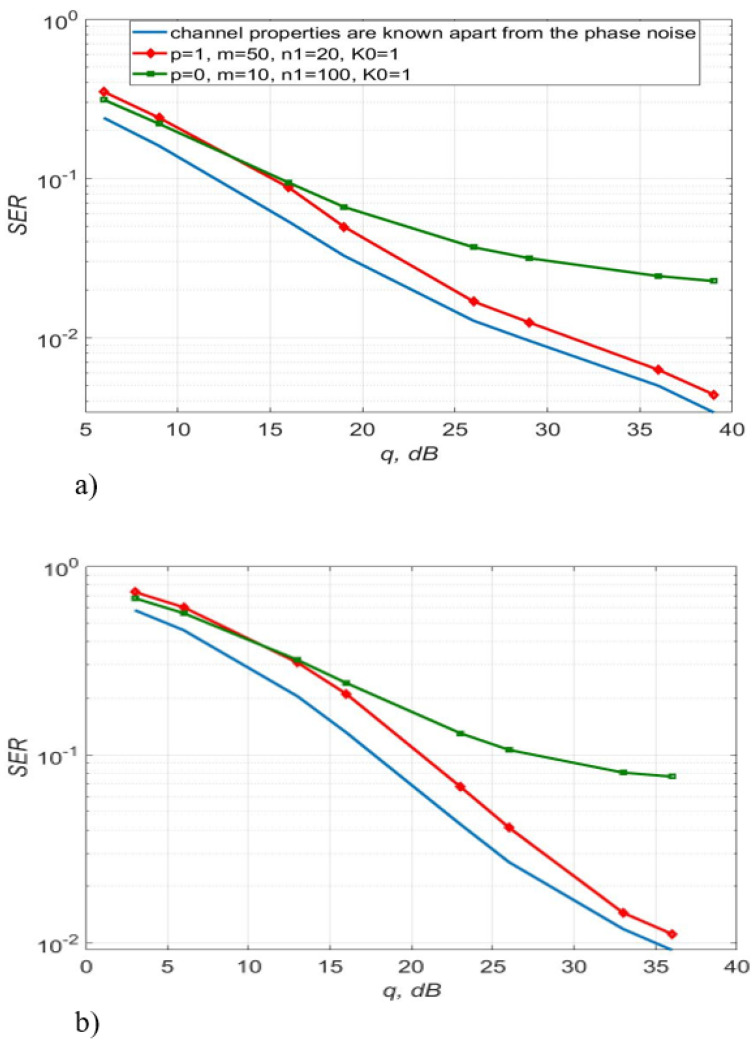
SER of 16-QAM—(**a**), 64-QAM—(**b**) versus SNR for the algorithms, described by Equations (5)–(9) and (12) with polynomial approximation of zero and the first order.

**Figure 7:**
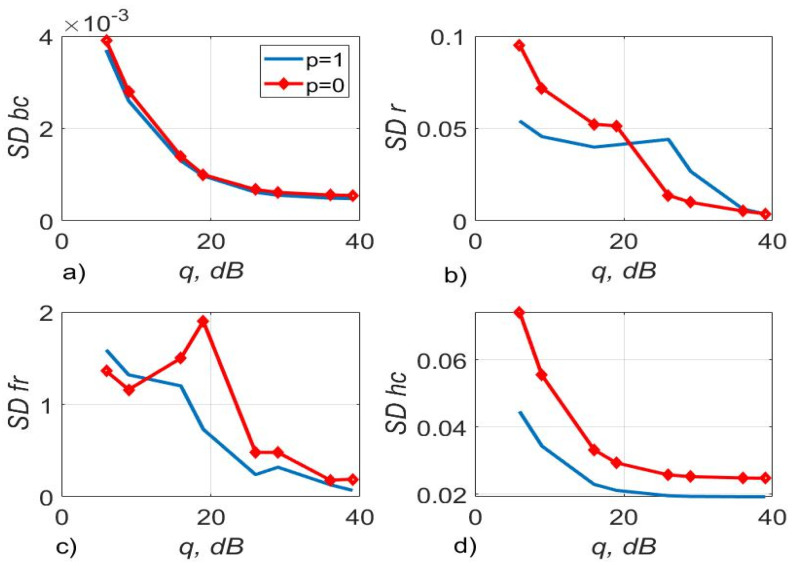
The SD of estimating DC offset—(**a**), amplitude imbalance—(**b**), phase imbalance (deg)—(**c**), and channel gains—(**d**) versus SNR per bit, the test signal was modulated by 16-QAM and the algorithms, described by Equations (5)–(9) and (12) with p=1, p=0 was used.

**Figure 8:**
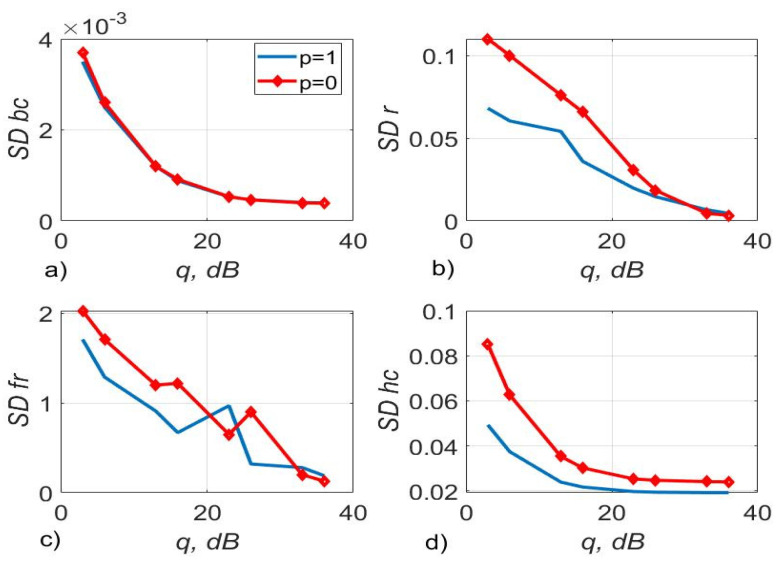
The SD of estimating DC offset—(**a**), amplitude imbalance—(**b**), phase imbalance (deg)—(**c**), and channel gains—(**d**) versus SNR per bit, the test signal was modulated by 64-QAM and the algorithms, described by Equations (5)–(9) and (12) with p=1, p=0 was used.

**Figure 9:**
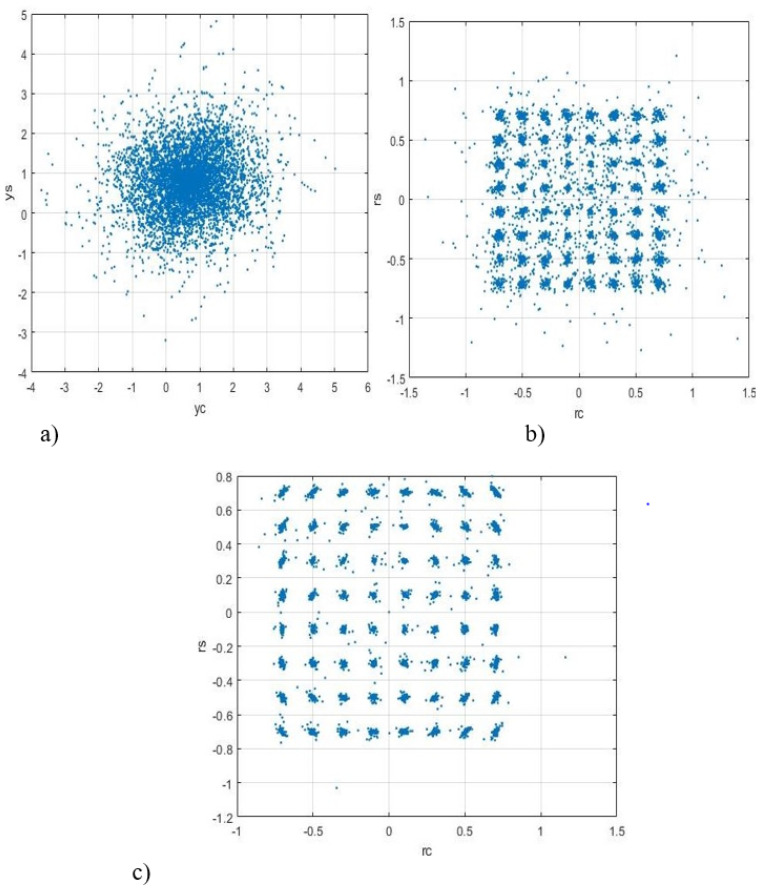
Constellations of the 64-QAM (SNR is 26 dB per bit) at the input of the compensator—(**a**); at the output of the compensator which uses the algorithms, described by Equations (5)–(9) and (12) with $p=0,\;m=10,\;n_{1}=100,\;K_{0}=1$—(**b**); $p=1,\;m=50,\;n_{1}=20,\;K_{0}=1$—(**c**).

**Figure 10:**
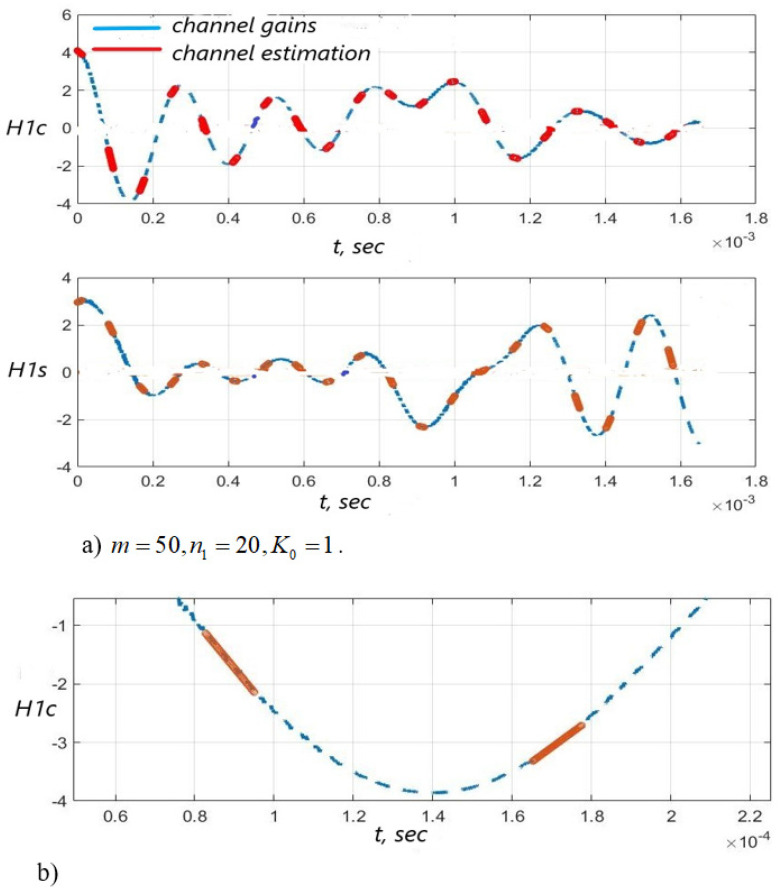
Channel gains $H_{1c}(i),\;H_{1s}(i)$ and their estimation based on the test signal versus time: the algorithms, described by Equations (5)–(9) and (12) with p=1 was used—(**a**), SNR = 26 dB; the enlarged fragment of “a)” part of the Figure—(**b**).

**Figure 11:**
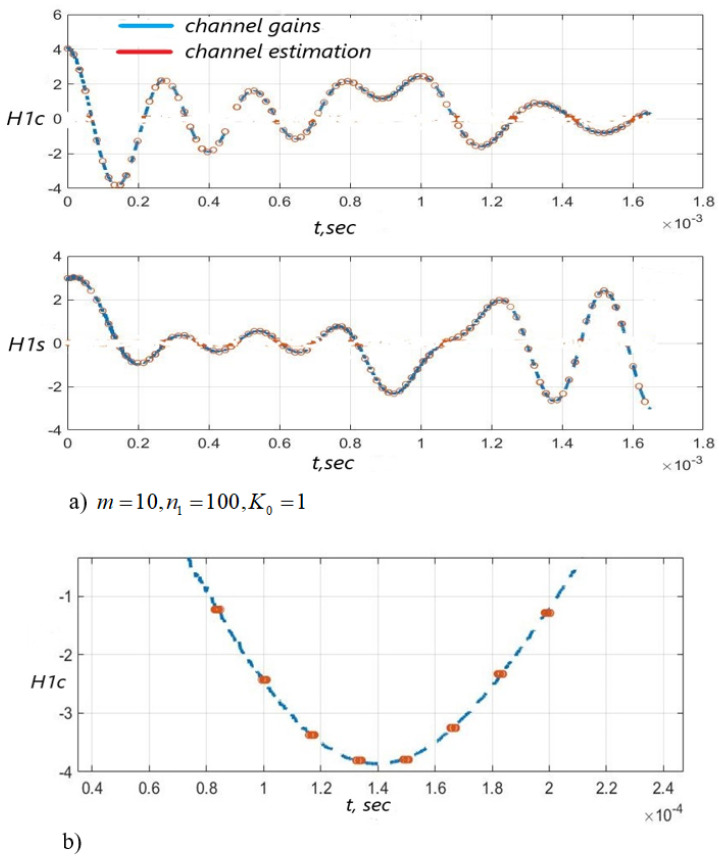
Channel gains $H_{1c}(i),\;H_{1s}(i)$ and their estimation based on the test signal versus time: the algorithms, described by Equations (5)–(9) and (12) with p=0 was used—(**a**), SNR = 26 dB; the enlarged fragment of (**a**,**b**).

**Figure 12:**
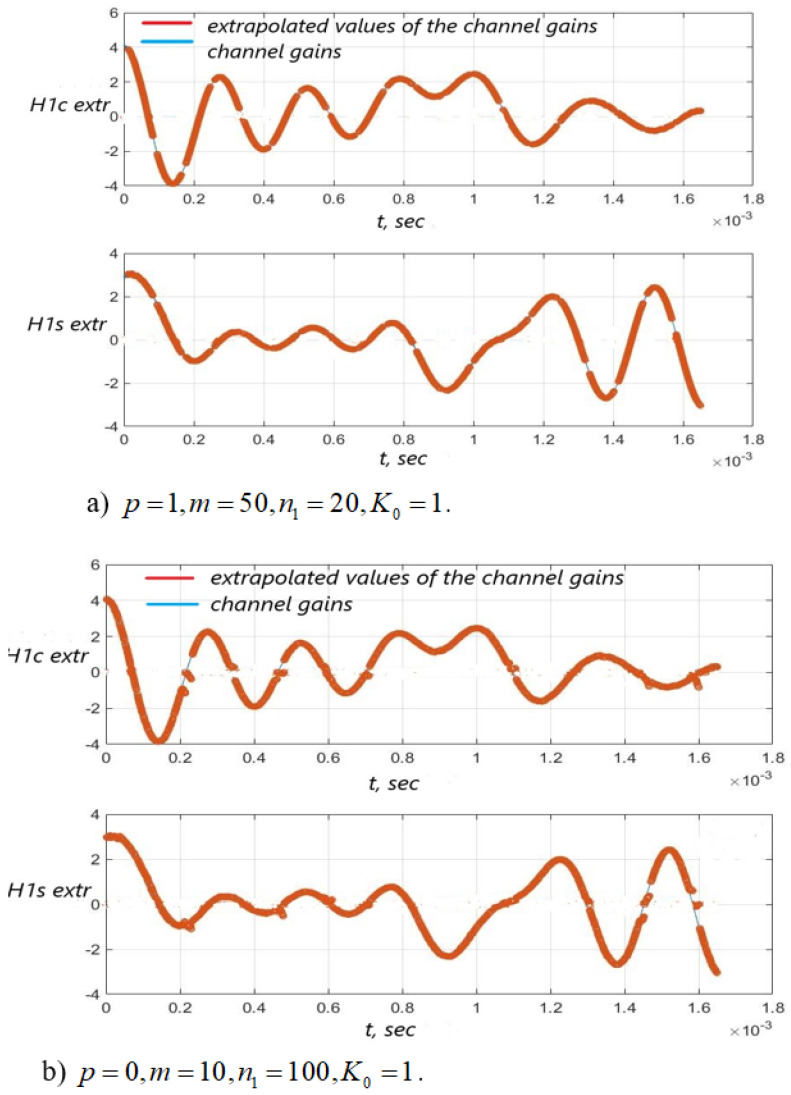
Extrapolated values of the channel gains $H_{1c}(i),\;H_{1s}(i)$ versus time for $(F_{D}+\Delta f)T=1.045\times 10^{-3}$ and p=1—(**a**), p=0—(**b**), SNR = 26 dB.

Figure 3, Figure 4 and Figure 5 show the dependence of the quality of the algorithms, described by Equation (5), (6) and (12), on the lengths of the test sequence m and the extrapolation interval $K_{0}$ for the signal 4-QAM. A polynomial approximation of the zero and first orders of the channel gains was used for this study. Figure 6 illustrates the noise immunity curves for receiving 16-QAM and 64-QAM. Figure 7 and Figure 8 show the SD of estimating unknown parameters of the communication channel and signal distortion in the direct conversion receiver. The test signal is modulated by 16-QAM and 64-QAM, respectively. Figure 9 shows constellations of the 64-QAM at the input of the receiver and at the output of the compensator which uses the algorithms, described by Equations (5)–(9) and (12) with a zero and first-order polynomial. The SNR was 26 dB per bit. Figure 10 and Figure 11 illustrate variations of the channel gains and their estimations over time for SNR of 26 dB per bit. Figure 12 shows the extrapolated values of the channel gains obtained using the Equations (5)–(9) with a zero and first-order polynomial.

Figure 3 shows that the selection of parameters m, $K_{0}$, $n_{1}$ makes it possible to obtain the least probability of signal reception error. Using a first-order polynomial approximation, this combination is $m=50,\;K_{0}=40,\;n_{1}=20$.

This can be easily seen from Figure 3 and Figure 4 that the best performance is for systems with characteristics $m=50,\;n_{1}=20,\;K_{0}=40$ for p=1 and $m=10,\;n_{1}=100,\;K_{0}=1$ for p=0. In this case, the energy gain is up to 5 dB for p=1 relative to the algorithm with p=0.

Figure 5 shows that the use of a first-order polynomial approximation allows obtaining a higher accuracy of estimation than using a zero-order polynomial. So, the standard deviation (SD) of estimating the amplitude imbalance is 2.8 times, the phase imbalance is 1.62 times, and the channel gains are 1.41 times less for the algorithm with p=1 than for the algorithm with p=0.

Figure 6 shows that the algorithms, described by Equations (5)–(9) and (12) with p=1 provides a higher noise immunity relative to the procedure with p=0. The first order of the polynomial makes it possible to obtain an energy gain of up to 10 dB for a 16-QAM signal and up to 12 dB for a 64-QAM signal relative to the algorithm using the zero-order polynomial.

Figure 7 shows that the use of (5)–(9), (12) with increases on average the accuracy of estimation of the phase imbalance by 1.65 times, channel gains by 1.41 times relative to the algorithm with for a 16-QAM signal.

Figure 8 shows that the use of (5)–(9), (12) with p=1 increases on average the accuracy of estimation of the phase imbalance by 1.25 times, channel gains by 1.42 times relative to the algorithm with p=0 for a 64-QAM signal.

Figure 9 shows a better compensation of signal distortions when the algorithms, described by Equations (5)–(9) and (12) with a polynomial of the first order is used relative to the approximation by a zero-order polynomial.

Also, Figure 10 and Figure 11 illustrate that the quality of the channel estimation is higher using a polynomial approximation with p=1, relative to the procedure with p=0. This is especially noticeable in Figure 10 and Figure 11b.

Figure 12 shows that the quality of the channel extrapolation is higher if the polynomial order is p=1.

Table 6, Table 7 and Table 8 show SER of 4, 16, 64-QAM in case of using different approximating structures in the algorithms, described by Equations (5)–(9) and (12).

Thus, for a channel with $(F_{D}+\Delta f)T=1.045\times 10^{-3}$, algorithms, described by Equations (5)–(9) and (12) with p=1 allows getting an energy gain of up to 5 dB for a 4-QAM signal and 10–12 dB for 16- and 64-QAM signals compared to the algorithm with p=0. The use of logarithmic and hyperbolic approximation does not bring benefits in the noise immunity of signal reception 4-, 16-, 64-QAM.

**Figure 13:**
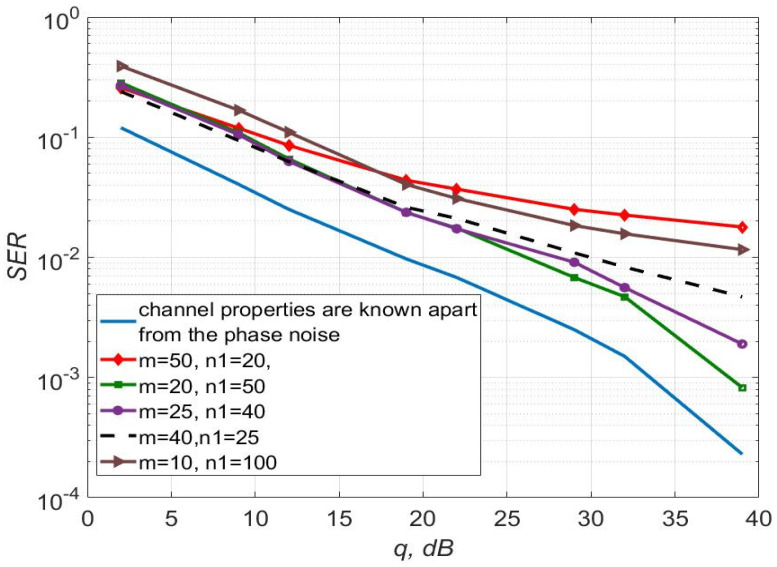
SER of 4-QAM versus SNR per bit; the algorithms, described by Equations (5)–(9) and (12) with p=1 was used for $K_{0}=1$ and different values of m and $n_{1}$.

Next, consider a communication channel for which $F_{D}=4$ kHz, Δf=10 kHz, T=0.25 µs, $(F_{D}+\Delta f)T=3.5\times 10^{-3}$. Figure 13 shows the noise immunity curves of 4-QAM for different lengths of the test sequence m and different numbers of transmission sessions $n_{1}$. It follows from Figure 13 that the best option is $m=20,\;n_{1}=50$. It gives an energy gain of 1 to 12 dB relative to the other considered options m and $n_{1}$.

**Figure 14:**
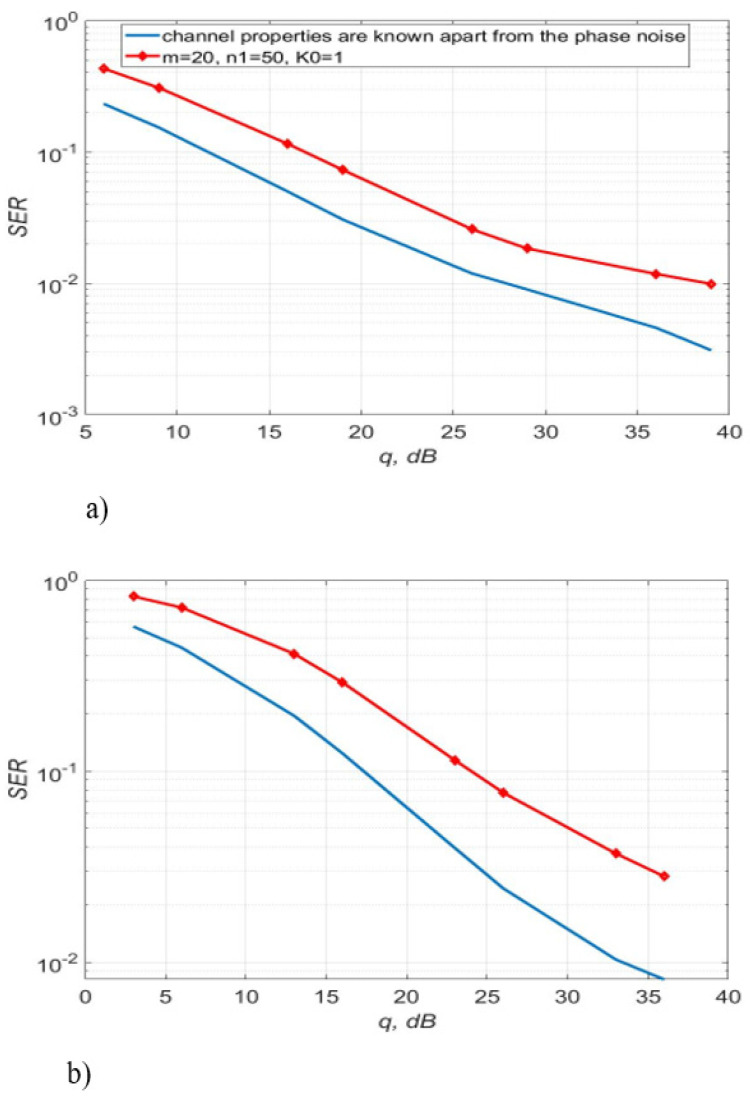
SER of 16-QAM (**a**), 64-QAM (**b**) versus SNR per bit: the algorithms, described by Equations (5)–(9) and (12), p=1.

**Figure 15:**
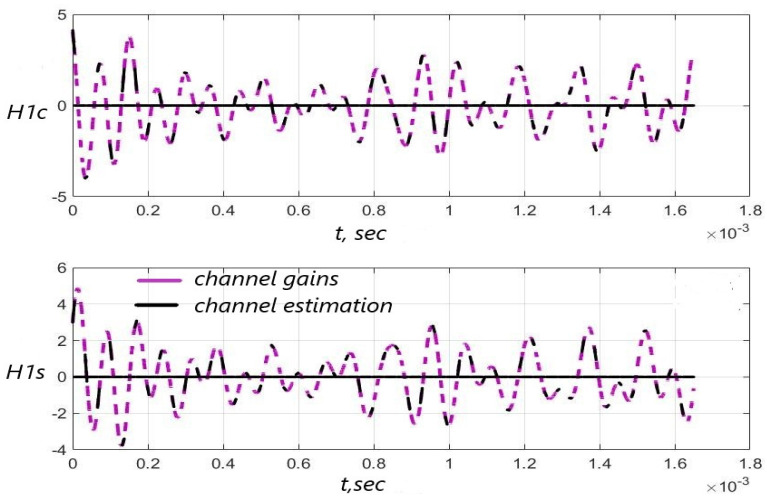
Channel gains $H_{1c}(i),\;H_{1s}(i)$ and their estimation based on the test signal versus time: $(F_{D}+\Delta f)T=3.5\times 10^{-3}$, p=1, SNR = 26 dB.

**Figure 16:**
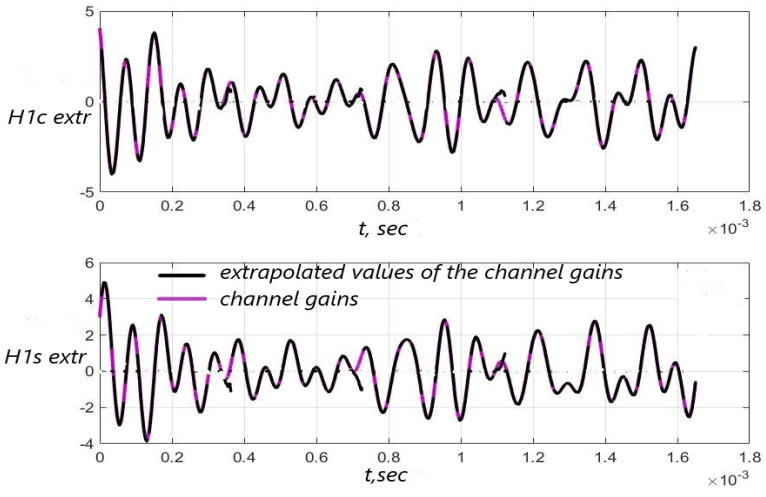
Extrapolated values of the channel gains $H_{1c}(i),\;H_{1s}(i)$ versus time for $(F_{D}+\Delta f)T=3.5\times 10^{-3}$ and p=1, SNR = 26 dB.

Figure 14 illustrates the reception quality of signals modulated by 16-QAM and 64-QAM. The first order of polynomial was chosen for simulation. Figure 15 and Figure 16 show the realization of the communication channel gains, as well as their estimations and extrapolated values obtained using the procedure (5)–(9), (12) with p=1. In this case, we used 64-QAM and SNR per a bit was 26 dB.

Figure 14 shows SER of 16, 64-QAM using algorithms, described by Equations (5)–(9) and (12) with a polynomial approximation of the first order. It can be seen that the phase noise and distortions of the direct conversion receiver lead to an increase in SER compared to the situation when only additive noise is taken into account. In addition, SER, instead of gradually decreasing, has an error floor at high values of SNR due to the phase noise and inaccurate distortion compensation. This effect is enhanced for a 64-QAM signal.

Figure 15 and Figure 16 show the performance of the channel estimation and extrapolation for a 64-QAM signal.

Therefore, the algorithms, described by Equations (5)–(9) and (12) allows getting the lowest SER for 4-QAM, 16-QAM, and 64-QAM for the channel with $(F_{D}+\Delta f)T=3.5\times 10^{-3}$ if the length of the test signal is 20, the number of transmission sessions is 50.

#### 3.2.2. MIMO Systems

We consider a channel with $(F_{D}+\Delta f)T=1.045\times 10^{-3}$. Polynomial, logarithmic, and hyperbolic functions with two parameters are taken as approximating structures for the communication channel gains in order to reduce the computational complexity of the estimation algorithms.

**Figure 17:**
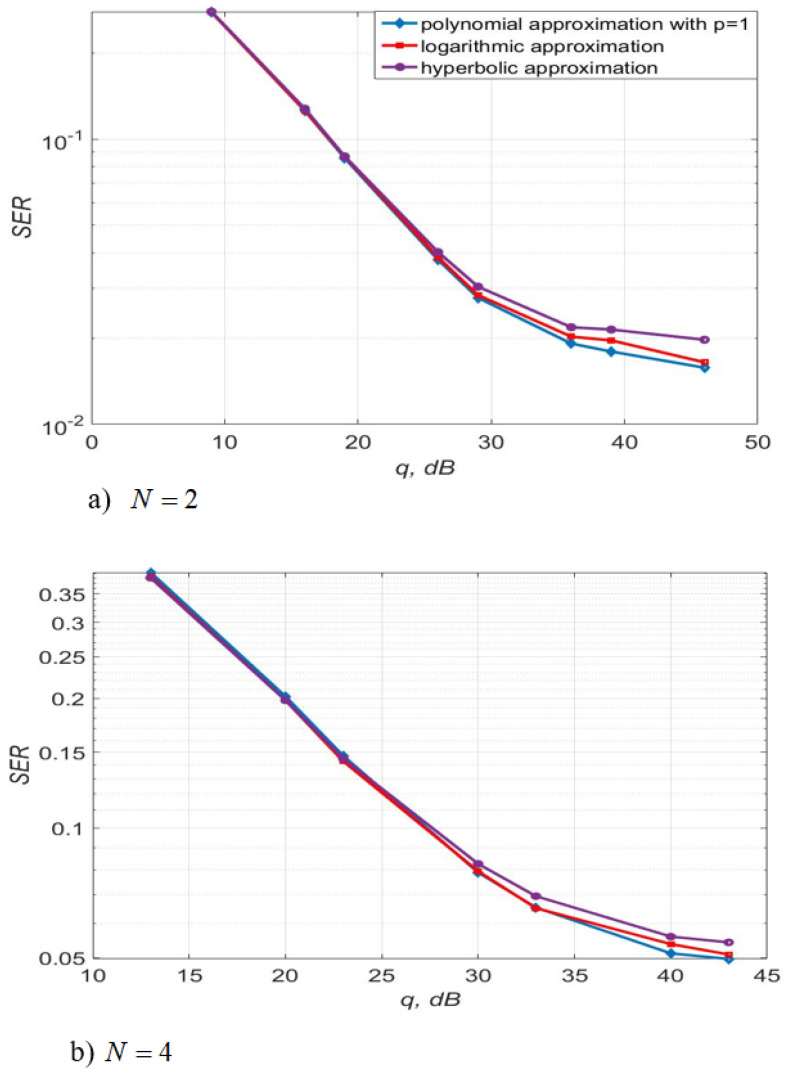
SER of 4-QAM versus SNR per bit for different approximating structures for MIMO systems.

**Figure 18:**
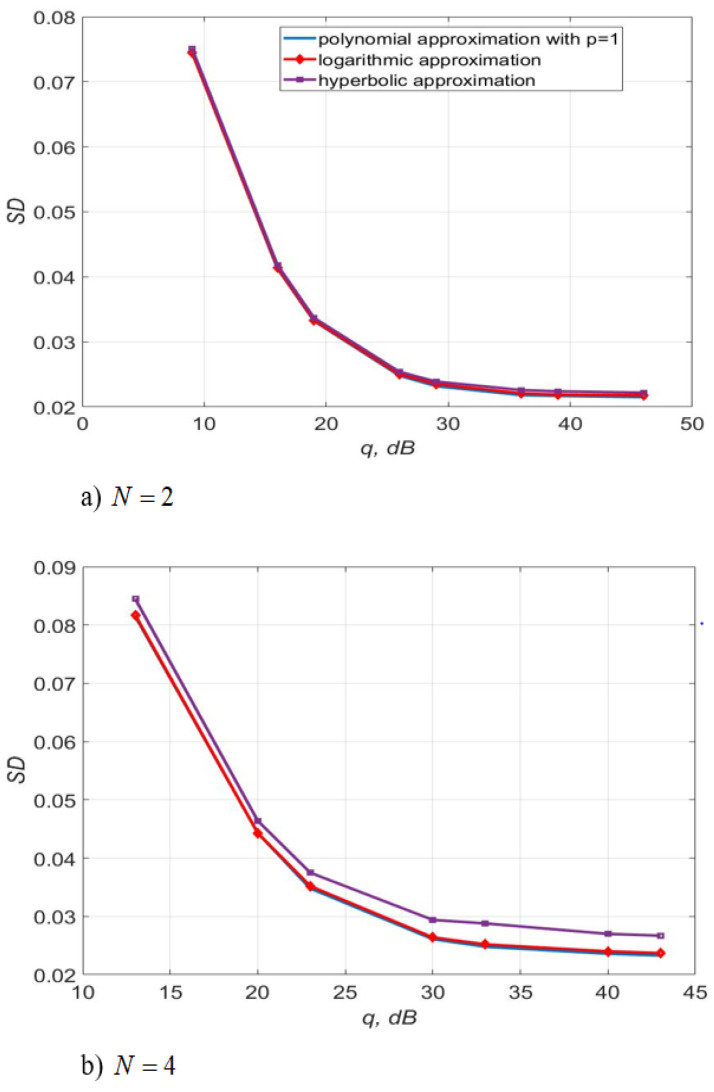
The SD of the channel gains estimation versus SNR per bit for different approximating structures for MIMO systems.

Figure 17 shows the 4-QAM reception immunity curves for a system with two and four transmit and receive antennas. Figure 18 illustrates the SD of estimating the communication channel gains for different values of SNR. Estimation of channel multipliers and distortions in the direct conversion receiver was carried out by the algorithms, described by Equations (5)–(9) and (12). The length of the test signal is m=50 symbols, the number of its transmission sessions is $n_{1}=20$, the length of the channel extrapolation and detection interval is $K_{0}=40$ symbols. The signal sample size is $m_{1}=90$.

Figure 17 demonstrates that the use of logarithmic and polynomial approximations of the first order leads to approximately the same 4-QAM signal reception noise immunity and to a small energy gain at large SNR relative to the hyperbolic approximation. For instance, the use of polynomial approximation provides an energy gain of 0.5–3 dB at SNR = 30–35 dB compared to the use of hyperbolic approximation with the same complexity of the algorithms.

Figure 18 demonstrates that the accuracy of the channel estimation is almost the same for all three considered types of approximation for N=2. For N=4 the estimation accuracy of the logarithmic and polynomial approximations is 1.3 times higher than the accuracy of the hyperbolic approximation.

## 4. Discussion

The choice of approximating functions for channel gains with a small number of estimated parameters is due to reducing the computational complexity of signal processing algorithms. This is especially true for multi-user systems, for example, for the Internet of Things. For a time-varying communication channel, the estimation task becomes more complicated, since it is necessary to introduce models that predict the state of the channel. The synthesized in the article algorithms are most efficient under conditions of a priori uncertainty about the channel statistics and noise distribution. This allows not carrying out channel identification, which also saves computing resources. Since the channel changes in time rather quickly, the application of a large number of antennas becomes more complicated. The larger N, the more difficult it is to estimate the channel with high accuracy. Since the estimation algorithm was designed for the case of the absence of a priori information about the channel statistics (for example, the correlation function), it has been used only for N = 1, 2, 4. However, it is planned to study the possibility of using N > 4 antennas in future works.

The algorithm for estimating non-stationary channel gains proposed in [31] is complicated (see Table 1) and requires a priori information about the channel. The computational experiment showed that the estimation algorithms, described by Equations (5)–(9) and (12) using the approximation in the form of a first-order polynomial outperforms the algorithm from [31]. So for a 64-QAM signal with SNR greater than 20 dB, an energy gain of up to 7 dB can be achieved (see Figure 2). In addition, the use of (5)–(9), (12) in this case allows estimating the channel less often than when we use the algorithm [31]. Thus, at $(F_{D}+\Delta f)T=1.45\times 10^{-4}$, the number of sessions of the algorithms, described by Equations (5) and (6) is 100 times less than in [31].

Obviously, the simplest approach is the approximation of the channel gains by a constant value over some short time interval, in which the channel is assumed to change slightly. This corresponds to a zero-order polynomial approximating construction (p=0). By adjusting the length of the test sequence m, the number of transmission sessions $n_{1}$, and the length of the extrapolation interval $K_{0}$, it is possible to reduce the probability of a reception error. For instance, if we consider SISO systems the best choice is $m=10,\;n_{1}=100,\;K_{0}=1$. Nevertheless, as shown by the computational experiment, the algorithms, described by Equations (5)–(9) and (12), using a polynomial approximation of the first 5 dB for 4-QAM and 12 dB for 16-QAM and 64-QAM, compared to the algorithm with p=0 (see order (p=1) and $m=50,\;n_{1}=20,\;K_{0}=1$, allows you to get an energy gain of up to Figure 4 and Figure 6). For 4-QAM signal, the accuracy of the estimation of the amplitude imbalance is 2.8 times, the phase imbalance is 1.62 times and the channel gain is 1.41 times higher using a polynomial of the first order than zero in the estimation algorithm.

The use of three approximating functions, such as first-order polynomial, logarithmic and hyperbolic, with the same computational complexity of the estimation algorithms, leads to almost the same noise immunity for the considered m, $n_{1}$, $K_{0}$ (SISO systems, see Table 2, Table 3 and Table 4). For MIMO systems N=2,4, the use of a first-order polynomial and logarithmic approximation in algorithms, described by Equations (5)–(9) and (12) makes it possible to slightly increase the accuracy of estimating the channel gains relative to the hyperbolic approximation. In addition, this approach allows the improvement of the noise immunity (see Figure 17 and Figure 18) compared to a logarithmic and hyperbolic approximation. Thus, the SD of the estimation of the channel gains is 1.3 less for the algorithm with polynomial and logarithmic approximation relative to the hyperbolic one. The use of polynomial approximation at SNR of 30–35 dB makes it possible to obtain an energy gain of 0.5–3 dB compared to the use of hyperbolic approximation with the same complexity of the algorithms.

## 5. Conclusions

Finally, we can summarize the main results:

(1) The proposed algorithms, described by Equations (5)–(9) and (12) was obtained under conditions of a priori uncertainty about the distribution of noise and the statistical characteristics of the communication channel. (2) Approximation constructions such as first-order polynomial, logarithmic, and hyperbolic for the considered communication channel models for SISO systems have quite the same estimation quality. The best approximation with the minimum number of estimated parameters for MIMO systems is a polynomial of the first order and logarithmic approximation. (3) It is possible to choose the length of the test signal and the number of sessions of its transmission, in which the minimum SER will be provided. (4) The assumption that the channel gains do not change for even a short time (using a zero-order polynomial) results in a loss in receive immunity. (5) The proposed estimation algorithms can be implemented using modern DSPs, and for compensation, a number of companies produce high-precision (with high linearity) IQ demodulator microcircuits, which provide for the possibility of supplying a digital code to compensate for conversion errors. For example, in the LTC5594 chip from ANALOG DEVICES. The LTC5594 contains circuitry for minimizing receiver impairments such as DC offset, phase and gain error, and nonlinearity. The gain error and phase error adjust, DC offset adjust, and nonlinearity adjust registers are digitally controlled through a four-wire serial interface.

## Figures and Tables

**Table 1:** Symbols, vectors, and matrices used in this work.

Notation of Symbols and Matrices	Description
${\mathbb{R}}^{N},{\mathbb{R}}^{2N}$	N(2N)—dimensional real vector space
E(⋅)	Expected value
N	Number of transmitting and receiving antennas
k	The number of the transmitting antenna.
l	The number of the receiving antenna.
i	Discrete time
$Y_{c}(i),Y_{s}(i)$	In-phase and quadrature components (IQ) of the received signal
$y_{c,l}(i),y_{s,l}(i)$	Elements of vectors $Y_{c}(i),Y_{s}(i)$
$H_{1}(i),H_{2}(i)$	In-phase and quadrature components of the channel matrix
$H_{1c,lk}(i),H_{1s,lk}(i)$	Elements of the matrix $H_{1}(i)$
$H_{2c,lk}(i),H_{2s,lk}(i)$	Elements of the matrix $H_{2}(i)$
$B_{c}(i),$ $B_{s}(i)$	IQ vectors of the DC offset
$b_{cl},b_{sl}$	Elements of vectors $B_{c}(i),$$B_{s}(i)$
Θ(i)	The vector of information symbols M-QAM or symbols of the test signal
$I_{k}(i),\;J_{k}(i)$	Elements of the vector Θ(i)
d(i)	The row vector the form of which is determined by the type of the approximation
$X_{1c,lk},\;X_{1s,lk}$	Vectors of coefficients of approximation of elements $H_{1c,lk}(i),H_{1s,lk}(i)$
$a_{1c0,lk},a_{1c1,lk}$, $a_{1s0,lk},a_{1s1,lk}$	Elements of vectors $X_{1c,lk},\;X_{1s,lk}$
$X_{2c,lk},\;X_{2s,lk}$	Vectors of coefficients of approximation of elements $H_{2c,lk}(i),H_{2s,lk}(i)$
$a_{2c0,lk},a_{2c1,lk}$,$a_{2s0,lk},a_{2s1,lk}$	Elements of vectors $X_{2c,lk},\;X_{2s,lk}$
$A_{\upsilon },B_{\upsilon },C_{\upsilon },D_{\upsilon },\upsilon =1,\ldots ,p$	Harmonic amplitudes in the trigonometric approximation from [31]
p	Approximation order
m	The length of the test signal
$n_{1}$	Number of test signal transmission sessions
$K_{0}$	The length of the channel extrapolation interval

**Table 2:** Parameters of the channel and signal distortion in the direct conversion receiver.

Parameter	Description
$\gamma_{l}$	Amplitude imbalance between IQ components at the l-th receiving antenna
$\Delta \varphi_{l}$	Phase imbalance between IQ components at the l-th receiving antenna
$b_{cl},b_{sl}$	DC offsets of IQ components at l-th receiving antenna
Δf	The frequency offset after demodulation
$h_{c,l\;k}(i),\;h_{s,l\;k}(i)$	Time-varying channel gains
$\varphi_{l}(i)=\varphi_{l0}+\zeta_{l}(i)$	Phase of the signal
$\zeta_{l}(i)$	Phase noise
$\varphi_{l0}$	An initial random phase
$T_{c}$	The duration of signal symbols $I_{k}(i),\;J_{k}(i)$ M-QAM

**Table 3:** Calculation of the computational complexity of the algorithm [31] for SISO systems.

Calculated Member	Number of Operations
V(i)F(i)	24(p+1)
$P(i)=\Gamma (i-1)+\sigma_{\zeta }^{2}{Ι}_{4(p+1)\times 4(p+1)}$	4(p+1)
$P(i){\left[V(i)F(i)\right]}^{T}$	8(p+1)(8(p+1)−1)
$V(i)F(i)P(i){\left[V(i)F(i)\right]}^{T}$	24(p+1)−3
$V(i)F(i)P(i){\left[V(i)F(i)\right]}^{T}+\sigma_{\mu }^{2}{Ι}_{2\times 2}$	2
${\left[V(i)F(i)P(i){\left[V(i)F(i)\right]}^{T}+\sigma_{\mu }^{2}{Ι}_{2\times 2}\right]}^{-1}$	8
$K(i)=P(i){\left[V(i)F(i)\right]}^{T}{\left[V(i)F(i)P(i){\left[V(i)F(i)\right]}^{T}+\sigma_{\mu }^{2}{Ι}_{2\times 2}\right]}^{-1}$	24(p+1)
Γ(i)=P(i)−K(i)V(i)F(i)P(i)	$64{\left(p+1\right)}^{2}$
$Y(i)-V(i)F(i)\overset{⌢}{X}(i-1)$	16(p+1)
$K(i)(Y(i)-V(i)F(i)\overset{⌢}{X}(i-1))$	12(p+1)
$\overset{⌢}{X}(i)=\overset{⌢}{X}(i-1)+K(i)(Y(i)-V(i)F(i)\overset{⌢}{X}(i-1))$	4(p+1)
The algorithm performs $N_{ОП}=[128{\left(p+1\right)}^{2}+100(p+1)+7]m$ operations in m iterations	

**Table 4:** Calculation of the computational complexity of the algorithms, described by Equations (5), (8) and (9) for MIMO systems with N transmitting and receiving antennas.

Calculated Member	Number of Operations
$D_{c}{}^{T}D_{c}$	$\left[2(6p+1)N^{2}+(10p+3)N](m-1\right)$
$D_{c}{}^{T}{\bar{Y}}_{c,l},\;D_{s}{}^{T}{\bar{Y}}_{s,l},l=1,\ldots ,N$	2[4(p+1)N+1]N(m−1)
${\left(D_{c}{}^{T}D_{c}\right)}^{-1},\;{\left(D_{s}{}^{T}D_{s}\right)}^{-1}$	$2{\left(2N(p+1)+1\right)}^{3}$
$\begin{array}{l} {\overset{⌢}{Z}}_{1,l}={\left(D_{c}{}^{T}D_{c}\right)}^{-1}D_{c}{}^{T}{\bar{Y}}_{c,l};\;{\overset{⌢}{Z}}_{2,l}={\left(D_{s}{}^{T}D_{s}\right)}^{-1}D_{s}{}^{T}{\bar{Y}}_{s,l},\\ l=1,\ldots ,N \end{array}$	$2N[8N^{2}{\left(p+1\right)}^{2}+6N(p+1)+1]$
${\overset{⌢}{\gamma }}_{l},\Delta {\overset{⌢}{\varphi }}_{l},l=1,\ldots ,N$	[2m(9N+1)+2N]N
Total number of operations: $\begin{array}{l} N_{ОП}=[10(p+1)(1+2N)+8N-3]mN+\\ +[16N^{2}{\left(p+1\right)}^{3}+8N{\left(p+1\right)}^{2}(3+2N)-2(p+1)(4N-1)]N+12N^{2}+7N+2 \end{array}$	

**Table 5:** The computational complexity of the algorithms, described by Equations (5), (8) and (9), and from [31].

The Order of Approximation	Algorithms, Described by Equations (5), (8) and (9)	Algorithm from [31]
p=1	$N_{ОП}≅65m+297$	-
p=2	$N_{ОП}≅95m+795$	$N_{ОП}≅1459m$
p=3	-	$N_{ОП}≅2455m$
p=4	-	$N_{ОП}≅3707m$
p=5	-	$N_{ОП}≅5215m$

**Table 6:** SER of 4-QAM versus SNR per bit for $m=50,\;n_{1}=20,\;K_{0}=40$.

q, dB	2	9	12	19	22	29	32	39
Polynomial approximation, p=1								
SER	$2.02\times 10^{-1}$	$6.65\times 10^{-2}$	$3.99\times 10^{-2}$	$1.34\times 10^{-2}$	$9.9\times 10^{-3}$	$4.2\times 10^{-3}$	$2.5\times 10^{-3}$	$7.09\times 10^{-4}$
Logarithmic approximation								
SER	$2.0\times 10^{-1}$	$6.7\times 10^{-2}$	$4.04\times 10^{-2}$	$1.36\times 10^{-2}$	$1.0\times 10^{-2}$	$4.2\times 10^{-3}$	$2.6\times 10^{-3}$	$7.7\times 10^{-4}$
Hyperbolic approximation								
SER	$2.03\times 10^{-1}$	$6.77\times 10^{-2}$	$4.08\times 10^{-2}$	$1.36\times 10^{-2}$	$1.0\times 10^{-2}$	$4.3\times 10^{-3}$	$2.6\times 10^{-3}$	$8.23\times 10^{-4}$

**Table 7:** SER of 16-QAM versus SNR per bit for $m=50,\;n_{1}=20,\;K_{0}=1$.

q, dB	6	9	16	19	26	29	36	39
Polynomial approximation, p=1								
SER	$3.05\times 10^{-1}$	$2.4\times 10^{-1}$	$8.78\times 10^{-2}$	$4.97\times 10^{-2}$	$1.69\times 10^{-2}$	$1.25\times 10^{-2}$	$6.3\times 10^{-3}$	$4.4\times 10^{-3}$
Logarithmic approximation								
SER	$3.47\times 10^{-1}$	$2.4\times 10^{-1}$	$8.86\times 10^{-2}$	$5.06\times 10^{-2}$	$1.68\times 10^{-2}$	$1.25\times 10^{-2}$	$6.4\times 10^{-3}$	$4.4\times 10^{-3}$
Hyperbolic approximation								
SER	$3.48\times 10^{-1}$	$2.42\times 10^{-1}$	$8.9\times 10^{-2}$	$5.06\times 10^{-2}$	$1.71\times 10^{-2}$	$1.26\times 10^{-2}$	$6.4\times 10^{-3}$	$4.5\times 10^{-3}$

**Table 8:** SER of 64-QAM versus SNR per bit for $m=50,\;n_{1}=20,\;K_{0}=1$.

**q, dB**	3	6	13	16	23	26	33	36
Polynomial approximation, p=1								
SER	$7.3\times 10^{-1}$	$6.06\times 10^{-1}$	$3.08\times 10^{-1}$	$2.1\times 10^{-1}$	$6.77\times 10^{-2}$	$4.1\times 10^{-2}$	$1.45\times 10^{-2}$	$1.12\times 10^{-2}$
Logarithmic approximation								
SER	$7.29\times 10^{-1}$	$6.05\times 10^{-1}$	$3.07\times 10^{-1}$	$2.09\times 10^{-1}$	$6.87\times 10^{-2}$	$4.08\times 10^{-2}$	$1.46\times 10^{-2}$	$1.12\times 10^{-2}$
Hyperbolic approximation								
SER	$7.29\times 10^{-1}$	$6.06\times 10^{-1}$	$3.09\times 10^{-1}$	$2.12\times 10^{-1}$	$7.03\times 10^{-2}$	$4.15\times 10^{-2}$	$1.55\times 10^{-2}$	$1.11\times 10^{-2}$

## Data Availability

Not applicable.

## References

[1] Schenk T.C.W., Fledderus E.R., Smulders P.F.M. (2007). Performance analysis of zero-if MIMO OFDM transceivers with IQ imbalance. J. Commun..

[2] Maham B., Tirkkonen O., Hjorungnes A. (2012). Impact of Transceiver I/Q Imbalance on Transmit Diversity of Beamforming OFDM Systems. IEEE Trans. Commun..

[3] Aissa J., Qi S., Alouini M.-S. Analysis and compensation of I/Q imbalance in amplify-and-forward cooperative systems. Proceedings of the 2012 IEEE Wireless Communications and Networking Conference (WCNC).

[4] Mokhtar M. (2014). OFDM Opportunistic Relaying Under Joint Transmit/Receive I/Q Imbalance. IEEE Trans. Commun..

[5] Gokceoglu A. (2014). Energy detection under IQ imbalance with singleand multi-channel direct-conversion receiver: Analysis and mitigation. IEEE J. Sel. Areas Commun..

[6] Semiari O., Maham B., Yuen C. Effect of I/Q imbalance on blind spectrum sensing for OFDMA overlay cognitive radio. Proceedings of the 2012 1st IEEE International Conference on Communications in China (ICCC).

[7] Semiari O., Maham B., Yuen C. (2014). On the effect of I/Q imbalance on energy detection and a novel four-level hypothesis spectrum sensing. IEEE Trans. Veh. Technol..

[8] Pestryakov A.V., Khasyanova E.R. (2013). Analysis of compensation methods for non-ideal operation of quadrature converters of digital radio receivers. Electrosvyaz.

[9] Mirabbasi S., Martin K. (2000). Classical and Modern Receiver Architectures. IEEE Commun. Mag..

[10] Laskar J., Matinpour B., Chakraborty S. (2004). Modern receiver front-ends. Systems, Circuits, and Integration.

[11] Kalman R.E., Bucy R.S. (1961). New results in linear prediction and filtering theory. Trans. ASME J. Basic Eng..

[12] Kalman R.E. (1960). New approach to linear filtering and prediction problem. Trans. ASME J. Basic Eng..

[13] Stratonovich R.L. (1960). Application of the theory of Markov processes for optimal filtering of signals. Radio Eng. Electron..

[14] Shloma A.M. (1986). Indirect Method for Nonlinear Filtering of Markov Processes. Radio Eng. Electron..

[15] Dzhigan V.I. (2013). Adaptive Filtering of Signals: Theory and Algorithms.

[16] Bakushinsky A.B., Kokurin M.Y. (2015). Iterative methods of stochastic approximation for solving irregular non-linear operator equations. J. Comput. Math. Math. Phys..

[17] Zhang X. (2018). Parameter Estimation for Class A Modeled Ocean Ambient Noise. J. Eng. Technol. Sci..

[18] Van Thinh V., Tue H.H. Performance of 16QAM over Rayleigh fading channel in the presence of non-Gaussian noise. Proceedings of the 2013 International Conference on Advanced Technologies for Communications (ATC 2013).

[19] Tue H.H., Tran H.T., Nguyen T.D. On the influence of a class of non-Gaussian noise on M-PSK systems. Proceedings of the 2012 International Conference on Advanced Technologies for Communications.

[20] Long K.-K., Dang-Ngoc H., Do-Hong T. Improving iterated Extended Kalman Filter for non-Gaussian noise environments. Proceedings of the 2011 6th International Forum on Strategic Technology.

[21] Tri N.M., Tue H.H. Performance of filterbank multicarrier/offset quadrature amplitude modulation under non-Gaussian additive noise. Proceedings of the 2015 International Conference on Advanced Technologies for Communications (ATC).

[22] Vuong B.Q., Huynh H.T., Do H.N. Monte-carlo performance analysis of OFDM system in the presence of multi-path fading environment and non-Gaussian noise. Proceedings of the 2018 2nd International Conference on Recent Advances in Signal Processing, Telecommunications & Computing (SigTelCom).

[23] Kadri A. Non-coherent detection of weak M-ary chirp signals in non-Gaussian impulsive noise. Proceedings of the 2012 8th International Wireless Communications and Mobile Computing Conference (IWCMC).

[24] Verzhbitsky V.M. (2005). Fundamentals of Numerical Methods: High School.

[25] Xiong Y., Ning W., Zhongpei Z. (2018). An LMMSE-Based Receiver for Uplink Massive MIMO Systems with Randomized IQ Imbalance. IEEE Commun. Lett..

[26] Aziz M., Ghannouchi F.M., Helaoui M. (2017). Blind Compensation of I/Q Impairments in Wireless Transceivers. Sensors.

[27] Zhang W., de Lamare R.C., Pan C., Chen M. Joint TX/RX IQ Imbalance Parameter Estimation Using a Generalized System Model. Proceedings of the 2015 IEEE International Conference on Communications (ICC).

[28] Haq K.N. (2015). Correction and Compensation of I/Q Imbalance and Multipath Channel. Ph.D. Thesis.

[29] Chung Y.-H., Phoong S.-M. Joint estimation of I/Q imbalance and channel response for mimo OFDM system. Proceedings of the 15th European Signal Processing Conference (EUSIPCO 2007).

[30] Gappmair W., Koudelka O. CRLB and low-complex algorithm for joint estimation of carrier frequency/phase and I/Q imbalance in direct conversion receivers. Proceedings of the 2012 8th International Symposium on Communication Systems, Networks & Digital Signal Processing (CSNDSP).

[31] Gappmair W. (2013). Low-Complexity Estimation of Carrier and Imbalance Parameters in Direct Conversion Receivers. Adv. Electron. Telecommun..

[32] Chen-Jiu H., Racy C., Sheen W.-H. (2009). Joint Least Squares Estimation of Frequency, DC Offset, I-Q Imbalance, and Channel in MIMO Receivers. IEEE Trans. Veh. Technol..

[33] Darsena D., Gelli G., Iudice I., Verde F. (2021). Detection and blind channel estimation for UAV-aided wireless sensor networks in smart cities under mobile jamming attack. IEEE Internet Things J..

[34] Kreindelin V.B. (2009). New Methods of Signal Processing in Wireless Communication Systems.

[35] Chen Y. (2020). Channel Estimation with Pilot Reuse in IQ Imbalanced Massive MIMO. IEEE Access.

[36] Khan N.K., Ramachandra G.E. Compressed Sensing Algorithms for SISO-OFDM Channel Estimation. Proceedings of the 2020 International Conference on Emerging Trends in Information Technology and Engineering (ic-ETITE 2020).

[37] Khan I., Rodrigues J.J.P.C., Al-Muhtadi J., Khattak M.I., Khan Y., Altaf F., Mirjavadi S.S., Jun Choi B. (2019). A Robust Channel Estimation Scheme for 5G Massive MIMO Systems. Wirel. Commun. Mob. Comput..

[38] Sahoo M., Sahoo H.K. Adaptive Channel Estimation & Capacity Analysis for MIMO OFDM Communication in Urban and Sub- urban Environments Using Sparse Diffusion LMS Algorithm. Proceedings of the 2019 5th International Conference for Convergence in Technology (I2CT).

[39] Wang X. (2020). Pilot-Assisted Channel Estimation and Signal Detection in Uplink Multi-User MIMO Systems with Deep Learning. IEEE Access.

[40] Poborchaya N.E. (2021). Synthesis of an Algorithm for Estimating Signal Distortions in a Direct Conversion Receiver Based on Combining a Regularizing Procedure and a Nonlinear Filtering Method. J. Commun. Technol. Electron..

[41] Poborchaya N.E. DC-offset and IQ-imbalance estimation in the MIMO system. Proceedings of the 2017 Systems of Signal Synchronization, Generating and Processing in Telecommunications (SINKHROINFO).

[42] Maksimov S.Y., Poborchaya N.E. Estimation of a channel factors and signal distortions in the MIMO system with a direct transform receiver under the conditions of rayleigh fading and doppler frequency dispersion. Proceedings of the 2020 Systems of Signal Synchronization, Generating and Processing in Telecommunications (SYNCHROINFO).

